# Digital phenotyping for assessment and prediction of interoception, chronic stress, and self-regulation in adults: a scoping review

**DOI:** 10.3389/fdgth.2026.1710891

**Published:** 2026-02-09

**Authors:** Marta Alvarez-Ambrosio, Paloma Chausa, Diego Moreno-Blanco, Alba Roca-Ventura, Ignacio Oropesa, Gabriele Cattaneo, Patricia Sánchez-González, Javier Solana-Sánchez, Enrique J. Gómez

**Affiliations:** 1 Biomedical Engineering and Telemedicine Centre, ETSI Telecomunicación, Center for Biomedical Technology, Universidad Politécnica de Madrid, Madrid, Spain; 2 Instituto de Investigación Hospital 12 de Octubre (imas12), Hospital Universitario 12 De Octubre, Madrid, Spain; 3 Institut Guttmann, Institut Universitari de Neurorehabilitació Adscrit a la UAB, Barcelona, Spain; 4 Institut D'Investigació en Ciències de la Salut Germans Trias I Pujol, Badalona, Spain; 5 Centro de Investigación Biomédica en Red de Bioingeniería, Biomateriales y Nanomedicina, Instituto de Salud Carlos III, Madrid, Spain

**Keywords:** brain health, chronic stress, digital phenotyping, interoception, mental health, self-regulation, smartphone, wearable device

## Abstract

**Introduction:**

Digital phenotyping, the real-time quantification of human phenotype *in situ* via digital devices, offers opportunities to understand how behavior change interventions influence brain and mental health. Interoception, chronic stress, and self-regulation are key domains, benefiting from real-world, continuous assessment beyond what traditional methods can provide.

**Objective:**

The aim of this scoping review was to map and synthesize the literature of the last five years on the use of digital phenotyping to measure or predict interoception, chronic stress, and self-regulation in adults. We focused on the types of devices and sensors employed, the psychological domains targeted, the nature of the data collected, feature extraction, data processing methods, and technological platforms utilized.

**Methods:**

Following Joanna Briggs Institute methodology and PRISMA-ScR guidelines, we systematically searched PubMed, Web of Science, and Scopus, complemented with Google Scholar. Eligibility criteria included studies published since 2018, using smartphones or commercial wearables to assess or predict interoception, chronic stress, or self-regulation in adults.

**Results:**

From 850 retrieved records, 18 studies met inclusion criteria. Of these, 11 addressed chronic stress or stress reactivity, five self-regulation, and two interoception. Thirteen studies used wearable devices, three used smartphones, and two combined both approaches. Ecological momentary assessment (EMA) via smartphones was applied in eight studies. Heart rate variability (HRV) was the most common physiological measure (*n* = 14), followed by electrodermal activity and heart rate (*n* = 4 each). Nine studies analyzed behavioral data, including smartphone use, sleep, and activity. Six studies applied machine learning models, though only three reported classification accuracy (56.8%–79%). Eight used statistical methods to link features with stress or interoception, while four examined self-regulation using predefined features without identifying new biomarkers.

**Discussion:**

This review highlights that the field is still in its early stages, with most work focused on chronic stress and predominantly reliant on wearable devices. Integration of smartphone sensing and long-term monitoring remains limited, and analytical performance is modest. Nevertheless, the ubiquity of smartphones and wearables positions digital phenotyping as a promising, scalable approach for assessing brain and mental health in daily life. Future research should emphasize multimodal, longer-term data collection, innovative analytic methods, and transparent reporting.

## 1 Introduction

Neurological disorders have become the leading cause of illness and disability worldwide affecting over three billion people in 2021 (1). Increasing evidence indicates that certain lifestyle factors play a crucial role in these disorders. A large-scale study published in Lancet Neurology (2) analyzed twenty modifiable risk factors that could potentially influence the development of neurological diseases and concluded that some of these factors are key contributors. Another report from the Lancet Commission on dementia prevention (3) identified 12 modifiable risk factors for dementia, estimating that these factors account for around 40% of worldwide dementias, theoretically making them preventable or delayable. In both studies, risk factors included preventable lifestyle behaviours such as smoking, poor diet, high alcohol use, and low physical activity, among others.

Changing unhealthy behaviours is challenging and maintaining these changes long-term is even harder. This highlights the need for research into the mechanisms behind harmful behaviours and effective strategies to modify them, with growing interest in interventions that improve brain and mental health (4). Among the diverse mechanisms involved in health behaviour and brain health research, chronic stress, self-regulation and interoception have frequently been identified as key domains (5–10). Research in behavioral science indicates that stress and self-regulation are crucial mechanisms in facilitating behavior change, including adherence to interventions (11–13), and both domains are suggested as key research areas to uncover the mechanisms that underlie behavior change (14–17).

Stress arises when situations are perceived as exceeding one's resources (18). Acute stress is short-term and adaptive, whereas chronic stress produces harmful effects on body systems, including the nervous system (19–23). Prolonged activation of stress responses leads to allostatic load, the cumulative biological “wear and tear” from long-term exposure to stress (24) and is widely considered to increase vulnerability to metabolic, cardiovascular, and neurological disorders (25–29). Stress reactivity, whether exaggerated or blunted, has also been linked to different physical and mental health outcomes (30–32).

Self-regulation refers to the ability to modulate behavior in pursuit of long-term goals (33). It can encompass three main dimensions: emotion regulation, cognitive regulation, and self-related processes (such as self-affirmation and self-efficacy) (12). Deficits in self-regulation have been associated with impaired brain health, including difficulties in emotional control, decision-making, and impulse regulation (34, 35) possibly linked to alterations in neural circuits of executive function (35, 36), although the nature of this relationship is not fully understood. Compromised self-regulation increases vulnerability to stress, reduces cognitive flexibility, and elevates risk for mental health problems (34). Moreover, poor self-regulation contributes to the persistence of health-risk behaviors such as poor diet, physical inactivity, and substance use (14, 17).

Alongside stress and self-regulation, interoception (a relatively understudied domain in behavioral science), shows significant potential for understanding mechanisms of behavior change. Interoception refers to the process by which the nervous system senses, interprets and integrates signals originating from within the body, providing a moment-by-moment mapping of the body's internal landscape across conscious and unconscious levels (8). Interoceptive processing occurs across major biological systems that maintain homeostasis (8). Dysfunction in interoception has been linked to physical health problems, neuropsychological disorders, and emotional difficulties, and may represent a transdiagnostic vulnerability factor for psychopathology (8–10, 37).

Contemporary frameworks in affective neuroscience and psychology conceptualize interoception, stress and self-regulation as components of an integrated regulatory system that links physiological monitoring, stress responsivity, and behavioural control (38–42). Taken together, these integrative models and the empirical evidence outlined above offer a well-supported theoretical basis for examining these three domains jointly: their dynamic interactions shape vulnerability to health-risk behaviours, influence adaptation, and underpin key mechanisms of brain and mental health. This integrative perspective justifies their joint examination in the present scoping review.

Regardless of the approach taken to the design and deployment of a behaviour change intervention, a mayor issue is the operationalization of outcomes, targets, and mechanisms of behaviour change, by providing clear definitions and assays, if possible, going beyond classic self-report measures (15, 43). The growing availability of smartphones and wearable devices has recently driven increased investigation into continuous, ambulatory monitoring of physiological states and behavior.

The term digital phenotyping was introduced by Onnela, Torous, and colleagues in 2016 (44) and is defined as the moment-by-moment quantification of the individual-level human phenotype *in situ* using data from smartphones and other personal digital devices. Digital phenotyping leverages the potential of data that are automatically generated and aggregated by smartphones and wearable devices, to measure (or offer robust proxies for) human behaviour and function in both disease and health (45). Recent reviews support its use in healthcare (46–49), particularly in neurological research (50–53), showing that digital phenotyping is a powerful tool for studying and promoting brain and mental health. Relatedly, digital biomarkers are objective, quantifiable physiological and behavioral measures collected via portable, wearable, implantable, or digestible devices (54, 55). Digital phenotyping allows the identification of these biomarkers, providing proxies for underlying physiological and behavioral processes. It shows promise for preventing, diagnosing, monitoring, and treating neuropsychiatric and neurodegenerative disorders (45, 51, 53, 56), as well as for developing adaptive, real-time interventions, including biofeedback and neurofeedback strategies (57–60).

To better understand the current research landscape within this field, we conducted a preliminary search to identify existing reviews on the use of digital phenotyping to study chronic stress, self-regulation, and interoception in everyday life. No prior reviews were identified that focused specifically on self-regulation or interoception using this approach. Several recent reviews have examined the use of wearable devices and smartphones in stress research (61–65), and broader mental health reviews have included stress-related studies (46, 47, 49, 66, 67). However, most focused on acute stress, specific populations, or experimental contexts, without addressing chronic stress as it manifests in everyday life. Together, these findings underscore a clear gap in the literature: no existing reviews consider these three domains together or synthesize the evidence on their assessment through digital phenotyping in everyday life contexts. Our review addresses this gap by focusing on chronic stress, alongside the domains of self-regulation and interoception, using commercial wearables and smartphones in real-world contexts.

Given the limited availability of prior reviews, a scoping review methodology is particularly appropriate, as it allows for mapping key concepts, identifying and analyzing research needs, and providing an overview of emerging fields. It also supports future research, and serves as part of the preparatory work for a longitudinal study currently in development.

## 2 Methodos

**Figure 1:**
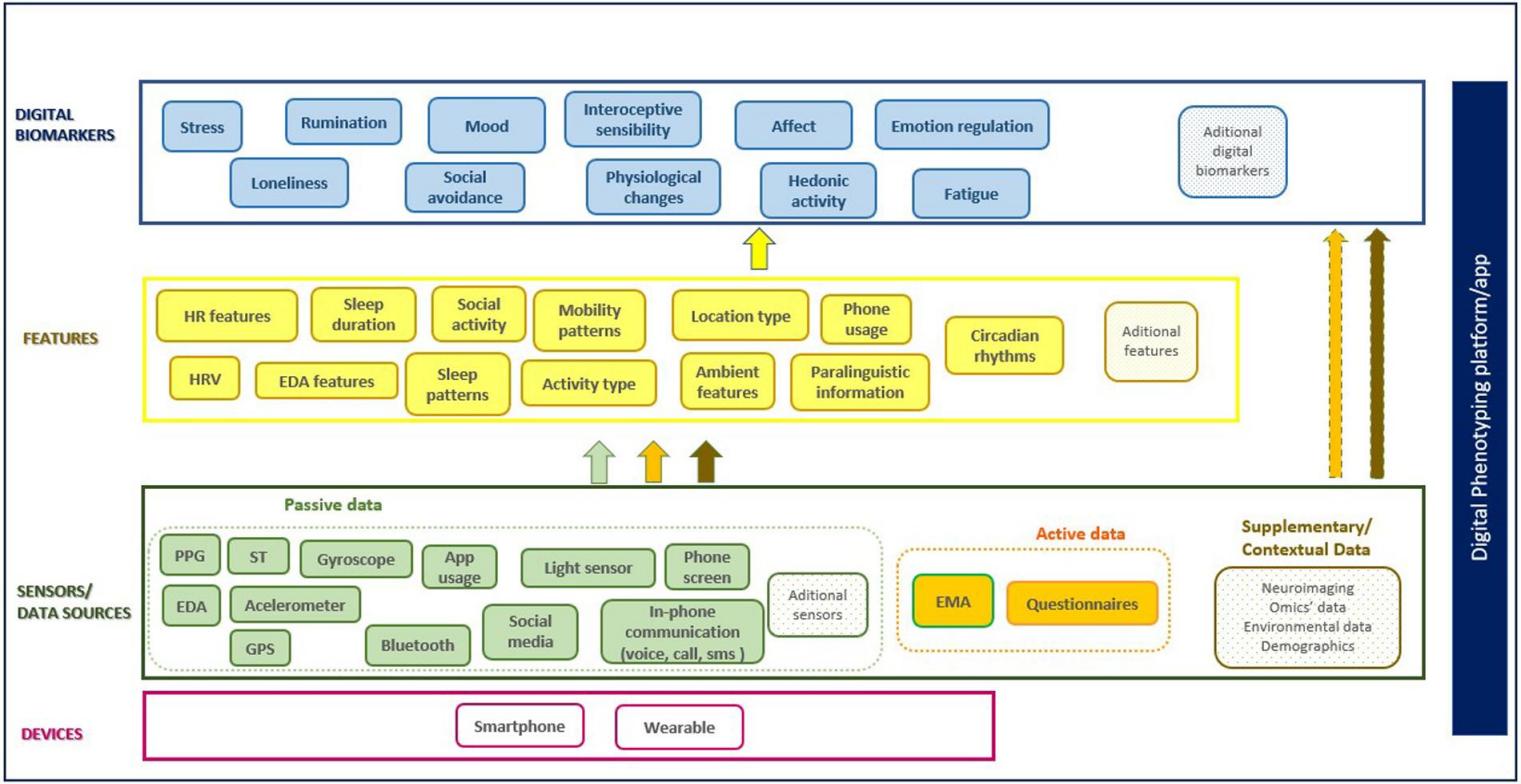
Digital phenotyping framework. The framework comprises five main components: (1) Devices, representing the hardware such as smartphones and wearables used for initial data acquisition; (2) Sensors/Data Sources, segmented into three distinct streams: passive data collected automatically from device sensors, active data requiring user engagement, and optional supplementary/contextual data for model enrichment; (3) Features, derived through signal processing and feature extraction techniques, which may independently function as digital biomarkers or require further analytical processing and integration with other streams; (4) Digital Biomarkers, the final layer of quantified constructs; and (5) Digital Phenotyping Platform/App, used for data collection and management. Adapted with permission from “Example of a layered, hierarchical sensemaking framework. Green boxes at the bottom of the figure represent inputs to the sensing platform” by David C. Mohr, Mi Zhang and Stephen M. Schueller, licensed under CC BY 4.0.

To structure the information from the studies and formulate the review questions, we have defined a digital phenotyping framework based on the model proposed by Mohr et al. (68), detailed in Figure 1. In this framework, data from various sources is captured and converted into features that provide meaningful information. These features can then be used to define digital biomarkers, often through machine learning (ML) or other analytical methods. In the end, the entire set of features and digital biomarkers can be used to identify clinical states and constructs. The framework also considers the technological platform or application used for data collection and management, which may also facilitate, among other things, the administration of questionnaires, self-reports, and informed consent management.

We applied the concepts of active and passive data as used by Torous et al. (69). Active data requires the subject's direct participation for generation, such as survey responses and Ecological Momentary Assessments (EMA), while passive data is generated without direct involvement from the subject, such as GPS traces or phone call logs. Additionally, the framework includes Supplementary/Contextual Data (e.g., neuroimaging, omics, or environmental data) for enrichment and contextual validation.

### 2.1 Review question

How is digital phenotyping used to measure and predict interoception, chronic stress, and self-regulation?

To fully address this overarching question, the following sub questions have been examined:

What types of devices (smartphones or wearables) and sensors are the studies using?
How do they operationalize domains and constructs?
What active and passive data do the studies use?
What features are used and what are they intending to measure?
What analytical methods are used to process and interpret digital phenotyping data?
What application/technological platforms are being used?
What are the main findings relevant to the domains examined in this scoping review?

This scoping review was conducted on the basis of the Joanna Briggs Institute (JBI) methodology for scoping reviews (70) and for the report (Methods) we have used the template that they propose (71). Furthermore, the review followed the Preferred Reporting Items for Systematic Reviews and Meta-Analyses extension for Scoping Reviews (PRISMA-ScR) checklist (72), shown in Supplementary Table S1.

In line with JBI guidelines for scoping reviews, no formal quality assessment of the included studies was performed.

The protocol used in this scoping review is detailed in the following sections.

### 2.2 Eligibility criteria

We applied the populations, concept, and context (PCC) framework to ensure the systematic inclusion of relevant studies and the exclusion of those not meeting the criteria.

#### 2.2.1 Population

This review focused on studies involving adult participants primarily aged 21–65 years. Studies that included participants slightly outside this range (starting at 18 or extending up to 70) were eligible as long as the majority of participants fell within the target age group. Animal studies were not considered.

#### 2.2.2 Concept

We included studies that examined the use of digital phenotyping as defined by Torous et al. (69), to assess or predict interoception, chronic stress, or self-regulation. Eligible studies met the following criteria: (1) they collected active and passive data using digital devices, potentially combined with external data sources (e.g., clinical records, neuroimaging); (2) the digital devices used were smartphones or wearable devices suitable for everyday use (e.g., wristbands, smartwatches, rings, or smart clothing); (3) they implemented analytical procedures aimed at extracting clinically or psychologically relevant insights. Studies relying exclusively on active data (e.g., EMA-only or digital diary–only designs) were excluded. Studies could additionally incorporate external data sources (e.g., medical records, neuroimaging), as long as digital phenotyping criteria were met.

#### 2.2.3 Context

Studies were eligible if they collected real-world (naturalistic) digital phenotyping data via smartphones or wearable devices. Studies conducted exclusively in laboratory, clinical, or institutional care settings were excluded unless they also collected real-world digital phenotyping data.

#### 2.2.4 Outcomes

Given the extensive literature on stress measurement, we restricted inclusion to studies explicitly reporting outcomes related to stress reactivity, emotional/affective reactivity, allostatic load, or chronic stress (as defined in the Introduction). For interoception and self-regulation, outcomes were considered in a comprehensive manner and could include, but were not limited to, interoceptive awareness, interoceptive accuracy, emotion regulation, self-control, or self-efficacy.

#### 2.2.5 Types of sources

We considered original peer-reviewed research papers, including experimental and quasi-experimental designs, analytical observational studies, qualitative studies, mixed-methods studies, and study protocols. Non-English publications were excluded due to resource limitations, as were reviews, meta-analyses, conference abstracts, editorials, opinion papers, and grey literature, since our objective was to analyze primary evidence and extract detailed information from original studies.

### 2.3 Search strategy

An initial limited search of PubMed and Web of Science was carried out to identify articles on the topic. Additionally, a selection of articles known to the authors was used to identify keywords and phrases related to this study. The text words contained in the titles and abstracts of relevant articles, and the index terms used to describe the articles were used to develop a full search strategy for PubMed (see Supplementary Table 2). The search strategy, including all identified keywords and index terms, was then adapted for all included databases (PubMed, Web of Science, and Scopus). Studies published since 2018 were considered to provide a contemporary overview of digital phenotyping research, reflecting recent advances in sensor accuracy, miniaturization, artificial intelligence integration, and connectivity (73, 74). Earlier literature may rely on outdated technologies no longer representative of the field's current potential.

A systematic search was conducted in the selected databases on October 23, 2023. The searches were rerun prior to the final analysis (in July 2024), in order to identify relevant studies published in the time frame between the first run and the final delivery of the scoping review.

### 2.4 Study selection

Following the search, all identified citations were collated and uploaded to RefWorks citation manager (Pro Quest LLC) and duplicates removed. After a pilot test, titles and abstracts were then screened by two independent reviewers for assessment against the inclusion criteria for the review. The full text of selected citations was assessed in detail against the inclusion criteria by the same two reviewers. Reasons for exclusion of sources of evidence at full text that do not meet the inclusion criteria were recorded. Any disagreements that arose between the reviewers were resolved through discussion.

### 2.5 Data extraction

One of the reviewers utilized a data extraction tool, specifically an Excel spreadsheet developed by the authors, to extract data from the papers. This extraction included detailed information about participants, concepts, contexts, study methods, and key findings pertinent to the review questions. The second reviewer subsequently verified the completed spreadsheet. The extraction form is available in the Supplementary Table S3.

### 2.6 Data synthesis

After data extraction, the included studies were synthesized using a descriptive and narrative approach. The extracted data, organized into a structured spreadsheet with categories such as study design, population characteristics, target domain, devices and sensors used, data types, pre-processing steps, analytical methods and key findings, were grouped according to review questions. Studies were primarily clustered by domain (interoception, chronic stress, and self-regulation) and further organized based on methodological aspects such as the types of digital devices used (smartphones, wearable sensors), the nature of active and passive data collected, preprocessing and feature extraction methods, and the analytical approaches applied (e.g., statistical methods or ML).

## 3 Results

**Figure 2:**
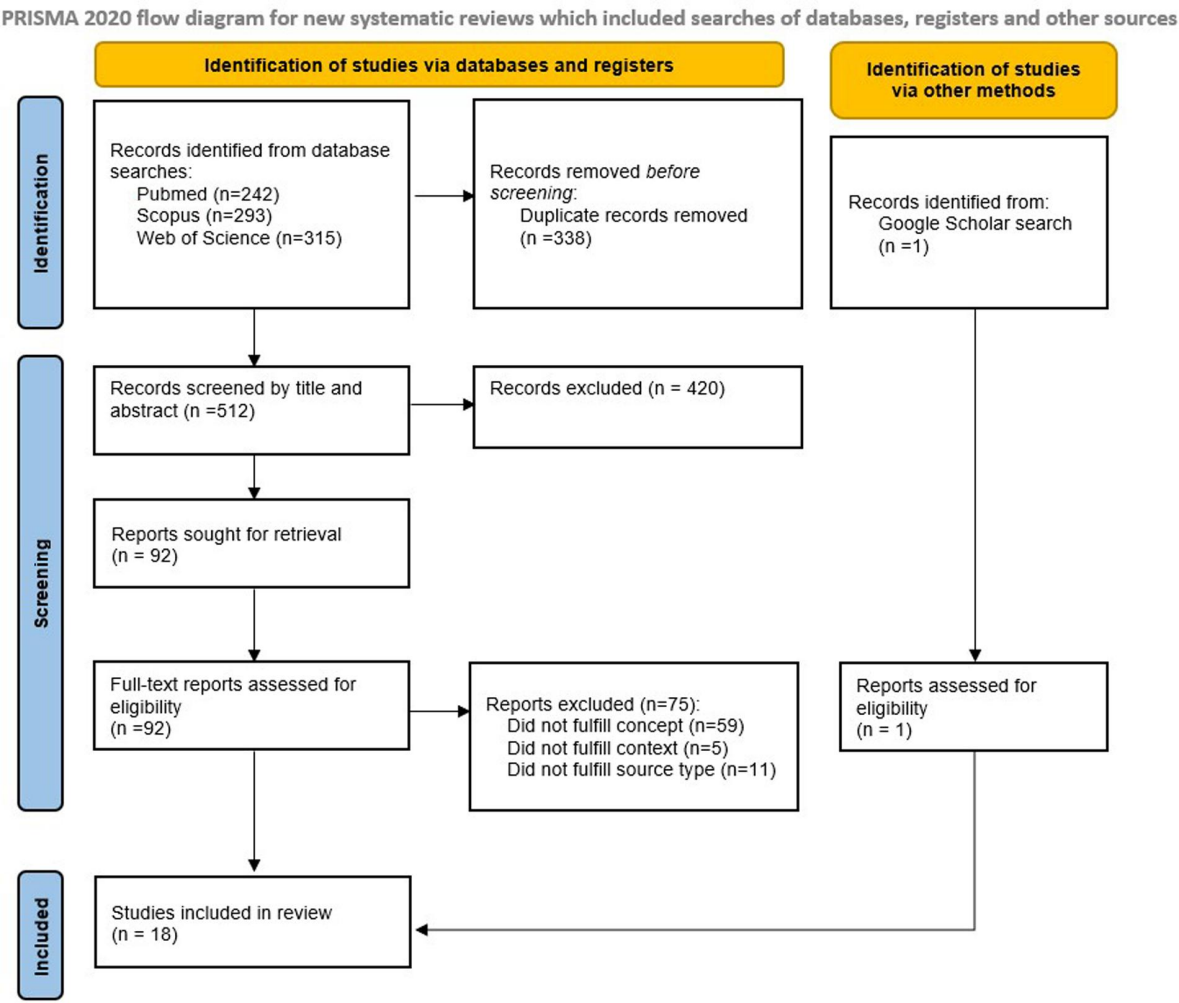
PRISMA flow diagram showing the identification and selection of studies.

The initial search yielded 850 papers. After removing duplicates, 512 studies were screened by title and abstract, resulting in 97 studies eligible for full-text review. Of these, 2 were not found and one was a conference poster. Additionally, the authors included one paper found through searches on Google Scholar. Finally, 18 eligible studies were identified. The search results and the inclusion and exclusion process are described in a PRISMA flow diagram (Figure 2).

### 3.1 Characteristics of the included studies

Of the 18 studies included, 11 addressed stress (75–85), 5 self-regulation (86–90) and 2 interoception (91, 92). Tables 1, 2 summarize the general information of the studies, including the sample population (size and type), purpose of the study, devices used, and key findings related to the review domains.

**Table 1:** Summary of the included studies on interoception and self-regulation.

Study Citation	Domain	Sample size & type (study length)	Study purpose	Devices	Main results
Kreibig et al. (86)	Self-regulation (emotion regulation)	237 Individuals with sleep bruxism	To phenotype ER in sleep bruxism with self-reported and physiological measures	Wearable (chest patch)	N/A (study protocol)
		(14 days)		Smartphone	
Vabba et al. (91)	Interoception	21 (a sub-sample from 245 participants of the study). Healthy participants	To assess interoception changes during COVID-19 stages and its relationship with HRV	Smartphone	HRV was positively associated with interoceptive accuracy before and during different stages of the pandemic
		(1 year)^a^			
Sharma et al. (87)	Self-regulation (emotion regulation)	31 Students	To explore the relationship between ER measures derived from wearable sensors and students' learning performance	Wearable (wrist-worn)	N/A (results do not apply to this review).
		(3 weeks)			
Plans et al. (92)	Interoception^b^	124 (1 day measurements were made for 120 s)	To develop and validate the Phase Adjustment Task, and examine the impact of physiological measures on task performance and interoceptive accuracy	Smartphone	HR, HRV and body mass index did not differ significantly between interoceptive and non-interoceptive participants
Schmid & Thomas (88)	Self-regulation	89 Health care professionals	To investigate the interaction of HRV and mindfulness (as correlates of self-regulation and well-being) from a within-person perspective	Wearable (chest patch)	N/A (results do not apply to this review)
		(1 week)			
Juarascio et al. (89)	Self-regulation (emotion regulation)	21 Individuals with clinically emotional eating behaviors	To test the hypothesis that momentary changes in HRV (as a trans-diagnostic biosignal of ER) can be used to detect risk of experiencing an emotional eating episode	Wearable (wrist-worn)	N/A (results do not apply to this review)
		(4 weeks)			
Williams et al. (90)	Self-regulation	100 adults with comorbid depression and obesity	To identify assays of self-regulation across various settings, examine their interrelations, and assess their mediating role in intervention adherence and mood and weight outcomes	Smartphone	N/A (study protocol)
		(2 years)			

^a^ The study lasted one year, with interoception-related measures collected at three time points: one laboratory session and two ambulatory sessions.

^b^ The paper comprises two studies, however only the first study is relevant to this review.

**Table 2:** Summary of the included studies on stress.

Citation	Domain	Sample size & type (study length)	Study purpose	Devices	Main results
Magal et al. (75)	Stress (chronic stress)	140 Healthy females	Predict chronic stress from physiological lifestyle and demographic features	Wearable (wrist-worn)	The model achieved 79% classification accuracy for chronic stress from a social tension source using a mixture of physiological (HR), lifestyle (activity, sleep) and demographic (smoking status) features
		(1 week)			
Tsujikawa et al. (76)	Stress (chronic stress)	168 Office workers (30 days data were collected for 30 days, the study lasted 10 months)	To develop an accurate chronic stress estimation system	Wearable (wrist-worn)	The classification system achieved 69.1% estimation accuracy in terms of increase/decrease in PSS with EDA and ACC features
Rodrigues et al. (78)	Stress (chronic stress)	5 Air traffic controllers	To evaluate chronic stress levels combining self-report measures with physiological biomarkers	Wearable (chest patch)	Findings reinforced the discriminatory power of AVNN and LF/HF for short-term stress classification using HRV measurements
		(7 days)			
Hirten et al. (77)	Stress (chronic stress)	361	To determine characteristics associated with longitudinal perceived stress and its relation with HRV	Wearable (smart watch)	N/A (results do not apply to this review)
		Health care workers			
		(5 months)			
van Kraaij et al. (79)	Stress (chronic stress)	328	To investigate the effect of chronic stress on HR over time while correcting for weekdays vs. weekends, and to test a possible modulation effect by gender and age	Wearable (chest patch)	The results showed a relationship between HR and the three-way interaction of chronic stress, gender, and the circadian harmonic
		Employees of technology companies			
		(5 days data was collected over 5 days, the study lasted 2 years)			
Schilling et al. (80)	Stress (stress reactivity)	201	To examine whether different levels of cardiorespiratory fitness differ about physiological stress reactivity and chronic stress	Wearable (chest patch)	Results were not consistent but showed lowered physiological stress reactivity (indexed by HRV) to acute work stress in officers with higher levels of cardiorespiratory fitness
		Police officers (2 days)			
Timmons et al. (81)	Stress (stress reactivity)	218	To investigate physiological stress reactivity as a factor contributing to the intergenerational transmission of aggression	Wearable (wrist-worn)	N/A (results do not apply to this review)
		Couples (1 day)			
Nakashima et al. et al. (82)	Stress (chronic stress)	64	To improve the performance of the early recognition of chronic stress, through more effective monitoring of physiological signals produced as people live their daily lives	Wearable (wrist-worn)	Results showed that the “activity state” approach was, to a statistically significant degree, superior to the “activity magnitude” approach in the recognition of chronic stress
		Healthy office workers			
		(1 month)			
Smets et al. (83)	Stress (stress reactivity)	1,002	To discover digital phenotypes of subjects' daily life stress responses to uncover blunted physiological responses to stress	Wearables (chest patch and wrist-worn)	Higher self-reported stress levels were associated with increase HR and decrease HRV; higher power of the phasic skin conductance component; a decrease of the skin temperature median; and blunted physiological stress responses
		Healthy office workers			
		(5 days per subject)			
				Smartphone	
Wilbur et al. (84)	Stress (chronic stress)	10	To clarify how sleep deprivation, poor sleep quality, and chronic stress affect participants' short- and long-term health	Wearable (sensorized shirt)	N/A (study protocol)
		Fishermen			
		(9 days)			
Berrocal & Katarzyna (85)	Stress (chronic stress)	(not specified)	To examine how peer feedback improves the accuracy and reliability of self-reported and sensor-based stress measures in natural settings	Wearable (wrist-worn)	N/A (study protocol)
		Healthy adults		Smartphone	
		(28 days)			

One study included 1,002 participants (83), while the remaining studies had sample sizes ranging from 5 to 361 participants, with 7 studies involving fewer than 100 participants (78, 82, 84, 87–89, 91). One study did not provide participant data (85). Three studies included participants with health conditions including sleep bruxism and mental health disorders (86, 89, 90) while the remaining studies included healthy participants.

Study durations varied, with the shortest study lasting one day (92) and the longest spanning 2 years (90). Interestingly, 83% of the studies (*n* = 15) had a duration of one month or less.

**Figure 3:**
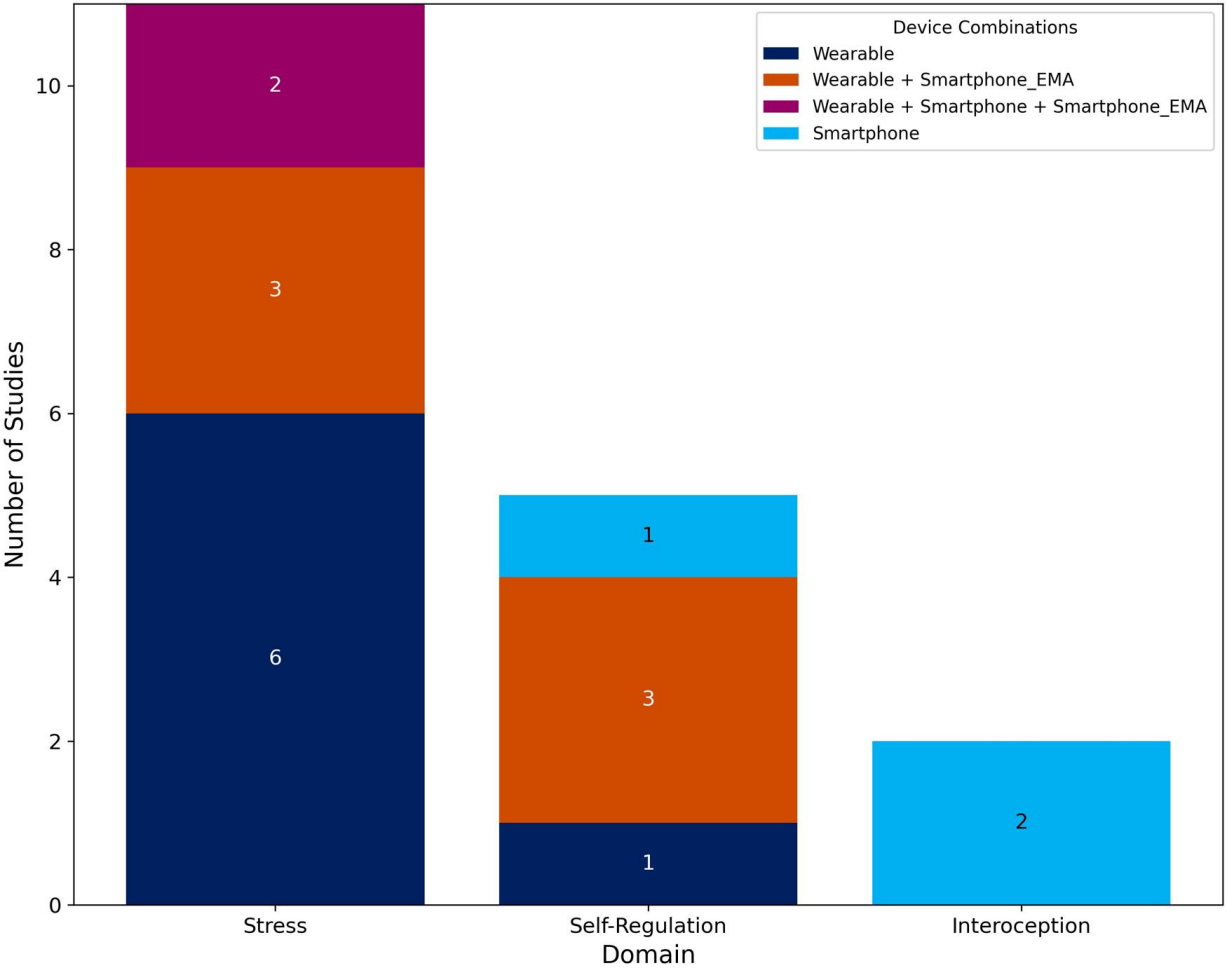
Device combinations by domain. This stacked bar chart summarizes the number of studies conducted in each of the three domains (*X*-axis): Stress, Self-Regulation, and Interoception. The *Y*-axis indicates the number of studies. Within each bar, the colored segments represent the specific device combinations used across studies, as detailed in the Device Combinations legend.

Of the 18 studies, 13 used wearable devices to collect passive data (75–82, 84, 86–89), 3 used a smartphone (90–92), 1 study used both a wearable device and a smartphone (85), and one study used 2 wearable devices and 1 smartphone (83) (Figure 3) The most widely used devices were wrist-worn devices, with 8 studies employing them (75, 76, 81, 82, 85, 87, 89), including a smartwatch (77). Six studies utilized electrocardiogram (ECG) patches (78–80, 83, 86, 88), and 1 study used a sensorized shirt (84). Wearable devices are classified as research-grade (RG) or consumer-grade (CG), as indicated in Table 3. Eight studies used smartphones for ecological momentary assessment (EMA) (77, 80, 81, 83, 85, 86, 88, 89). Supplementary Tables S4, S5, and S6 provide detailed information on the active and passive data used in the studies.

**Table 3:** Characteristics of the devices used in the studies.

Citation	Devices type, model, grade, smartphone	Sensors	App, Platform
Kreibig et al. (86)	Chest-patch	ECG, ACC	VivaLink cloud Qualtrics survey software and platform (for EMA)
	ECG VSUS4—ECG (VivaLink) (RG)		
	Smartphone (EMA)		
Vabba et al. (91)	Smartphone	Camera PPG	Cardiograph app (MacroPinch)
			HRV Camera app
			SurveyMonkey (Momentive Inc.) online questionnaires
Magal et al. (75)	Wrist-worn	ACC, altimeter, PPG	Fitbit cloud
	Charge 3 (Fitbit) (CG)		
Tsujikawa et al. (76)	Wrist-worn	EDA, ACC, skin temperature, gyroscope	Proprietary
	Embrace2 (Empatica) (RG)		
Sharma et al. (87)	Wrist-worn	PPG, skin temperature,	Not specified
	E4 (Empatica) (RG)	EDA, ACC	
Rodrigues et al. (78)	Chest patch	ECG, ACC^,^ skin temperature	Proprietary
	Vital Jacket technology (RG)		
Plans et al. (92)	Smartphone	Camera PPG	Custom app for Phase Adjustment Task
	iPhone 7 (Apple)	Barometer, ACC, gyro, proximity sensor, ambient light sensor, GPS	Online platform (Qualtrics) for questionnaires
Hirten et al. (77)	Smartwatch	GPS, altimeter, PPG, electrical heart sensor, ACC, gyroscope, ambient light	Apple Watch and Apple Health app
	Apple Watch (Apple)		
	Smartphone (EMA)		
Schmid et al. (88)	Chest patch	ECG, ACC, pressure, rotation, skin temperature	Movisens
	EcgMove3 and EcgMove4 (movisens) (RG)		Movisens*XS* app
	Smartphone (EMA)		
van Kraaij et al. (79)	Chest patch	ECG, ACC	Data were recorded on the devices' secure digital cards and uploaded to a secure internal cluster at the end of the experiment
	ECG (Gobio-Philips) (RG)		
Juarascio et al. (89)	Wrist-worn	PPG, skin temperature, EDA, ACC	Participants were instructed to upload data from their wristband to a Manager program on their computer at the end of each day.
	E4 (Empatica) (RG)		
	Smartphone (EMA)		
Schilling et al. (80)	Chest patch	ECG, ACC, pressure sensor	Movisesns
	ecgMove3 (movisens) (RG)		Movisens*XS* app
	Smartphone (EMA)		
Timmons et al. (81)	Wrist-worn	EDA, skin temperature, ACC	Survelytics app (EMA)
	Q sensor (Affectiva) (RG)		At the end of the study, data is downloaded onto a computer for processing
	Smartphone (EMA)		
Nakashima et al. (82)	Wrist-worn	PPG, skin temperature, EDA, ACC	Not specified
	Embrace2 (Empatica) (RG)		
Smets et al. (83)	Chest patch	ECG, ACC	At the end of the study, data is downloaded onto a platform for processing
	(Gobio—Philips) (RG)	Skin conductance, skin	
	Wrist-worn	temperature, ACC	
	imec's Chillband (RG)		Custom-made smartphone application for EMA
	Smartphone (sensors and EMA)		
Wilbur et al. (84)	Sensorized shirt	ECG, ACC, respiratory inductance plethysmography.	Every 12 h, the device is removed for the data to be uploaded to a database
	Hexoskin (Carre Technologies Inc) (RG)		
Williams et al. (90) Berrocal et al. (85)	Smartphone	GPS	Mindstrong app
	Wrist-worn	PPG, skin temperature, EDA, ACC	mQoL Lab app
	E4 (Empatica) (RG)		
	Smartphone (sensors and EMA)		

RG, research-grade, designed for scientific use.

CG, consumer-grade, designed for general consumer use.

Across the included studies, a variety of applications and technological platforms were used to collect and process active and passive data (Table 3). Some studies used platforms for EMA or surveys, such as Qualtrics (86, 92), SurveyMonkey (91), Survelytics (81), or custom EMA software (83). Others relied on multipurpose platforms capable of capturing both active and passive data, including mQoL Lab (85), Mindstrong (90) or Movisesns (80, 88). Additionally, some applications and platforms were dedicated to specific device or function, such as Fitbit (75), VivaLink (86), HRV Camera and Cardiograph mobile app (91) or Apple Health (77). In some studies, data were downloaded directly from the devices at regular intervals, or at the end of the monitoring period (79, 81, 83, 84, 89). One study used a proprietary solution (78). Finally, some studies did not explicitly specify the platforms or applications used for data collection, or the details were not fully outlined (76, 82, 87).

Regarding the 3 domains of this review, the physiological measure most frequently employed across all studies was heart rate variability (HRV), used in 12 studies (77, 78, 80, 83–89, 91, 92). Heart rate (HR) was utilized in 4 studies (75, 79, 83, 89) and electrodermal activity (EDA) was also used in 4 studies (76, 81–83). Additionally, 3 studies incorporated data on smartphone usage (83, 85, 90) and smartphone-based contextual measurements (83); One study examined sleep data (75), and 7 studies utilized activity or movement data (75, 76, 79, 82–84, 90), however, not all incorporated features derived from this data (activity) into their models. Some studies worked directly with raw sensor data, while others relied on pre-processed outputs provided by the wearable applications. This information was not consistently reported across studies and could only be noted when explicitly specified.

The conditions for device usage varied across the studies. Nine studies involved participants wearing the devices continuously in daily life (75, 77, 79–81, 83, 84, 86, 90), 3 studies required usage during work hours (76, 82, 88) and 2 studies employed the devices on a one-off basis (91, 92). In the remaining studies, participants used the devices under specific circumstances, such as during classes (87), waking hours (89), work shifts and days off (78) or twice daily (at wake-up and bedtime) (85).

The studies were categorized into three types based on their objectives and analytical methods: (1) those using ML models to classify and predict concepts related to the three domains explored in this scoping review (75, 76, 82, 83, 85, 90); (2) those investigating the relationship between features and domains (77–81, 84, 91, 92); and (3) those that, based on previous research, use features as measures associated with these domains (86–89). The first two types aim to find evidence that these models or features may represent relevant digital biomarkers.

**Figure 4:**
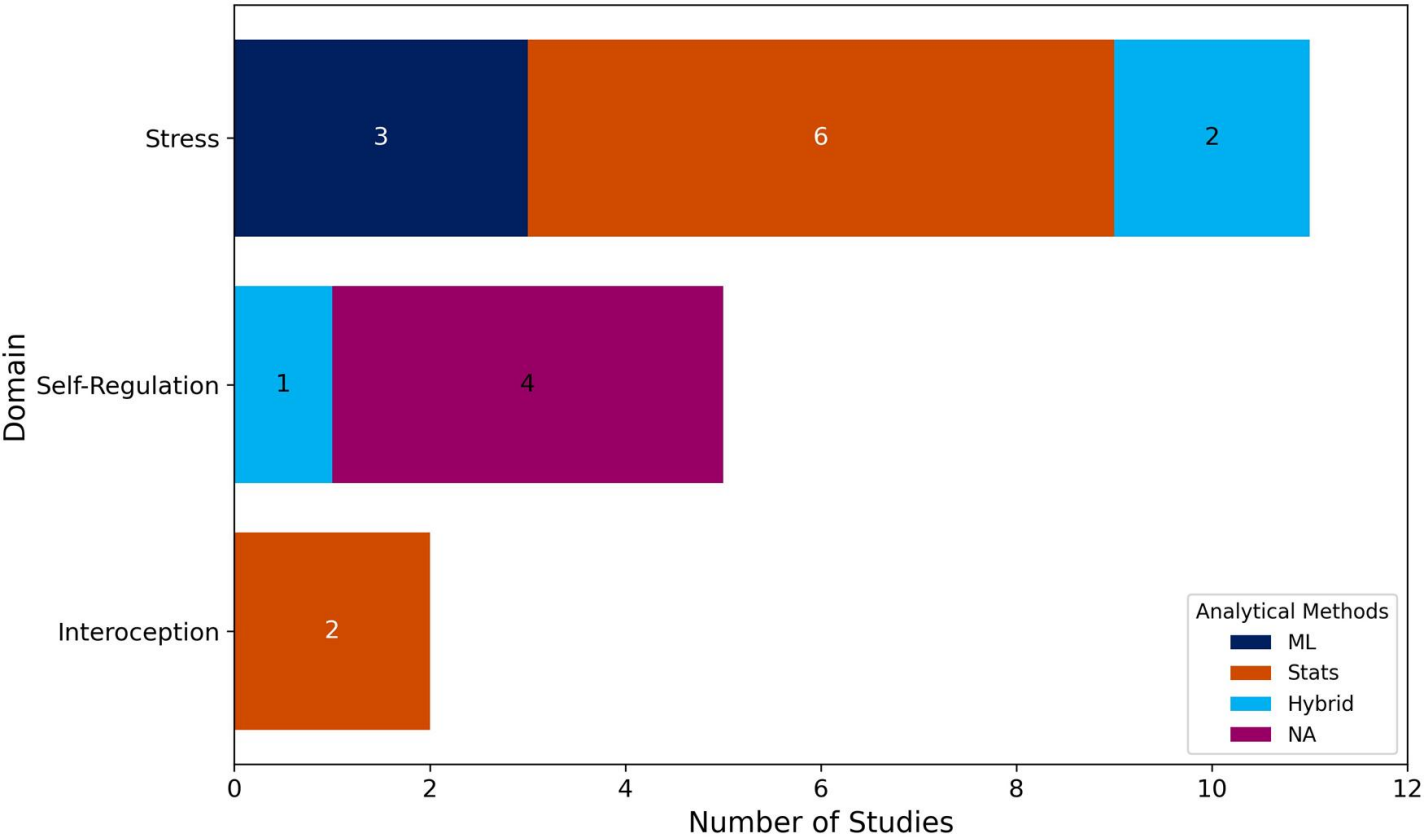
Analytical methods by domain. This stacked bar chart presents the analytical methods used across studies in each of the three domains (*X*-axis): Stress, Self-Regulation, and Interoception. The *Y*-axis indicates the number of studies. The colored segments within each bar correspond to the analytical method categories applied in the included studies, as specified in the Analytical Methods legend.

Of the 18 included studies, 4 were classified as Type 3, for which a methodological analysis does not apply in this review. Therefore, the methodological analysis focuses on the remaining 14 studies (Figure 4). Of these, 7 studies employed only statistical methods as their main analytical approach (77–81, 84, 91), while 7 incorporated ML methods, either through exclusively ML-based pipelines (75, 82, 85) or hybrid frameworks that combine statistical modeling with ML components (76, 83, 90, 92). Among the studies that employed statistical analysis methods, the most used approach was the Linear Mixed-Effects Model (LMM), which was applied in 4 studies. For ML approaches, supervised learning tasks, particularly regression and classification models such as Support Vector Regression (SVR) and Random Forest, were the most common (see details in Supplementary Tables S4, 5 and 6).

### 3.2 Description of the studies by domain

The results found for each domain (interoception, self-regulation, stress) are described below.

#### 3.2.1 Interoception

Of the 18 studies included in this scoping review, 2 examined measures of interoception (91, 92). The first study aimed to develop and test an interoceptive accuracy task (92). The other is a longitudinal study that explored changes in interoception and psychophysiological health and well-being during different stages of the pandemic in 2020 and assessed their potential association (91).

Both studies incorporated assessments of various interoceptive dimensions. They each measured interoceptive accuracy using different interoceptive tasks: the Phase Adjustment Task (PAT) (92) and the Heartbeat Counting Task (91). Furthermore, both studies used questionnaires (active data) to assess interoceptive sensibility. Nonetheless, the study by Plans et al. (92) did not explicitly refer to interoceptive sensibility, and instead discussed body perception and self-reported accuracy of the perception of interoceptive signals. For collecting physiological data, both studies used a smartphone with its camera to detect heartbeats via photoplethysmography (PPG).

Besides the findings from the interoceptive tasks, which classified individuals as interoceptors or non-interoceptors, both studies explored the correlation between different variables and task performance. The physiological evaluations in both studies focused on HRV metrics. Plans et al. (92) additionally considered body mass index (BMI) and psychological factors (depression, anxiety, stress, empathy, and self-reported interoceptive accuracy). The outcomes suggest that task performance is not affected by physiological or psychological variables. Conversely, in the research by Vabba et al. (91), scientists discovered that HRV, particularly mean RMSSD (root mean squares of successive difference), was positively linked to interoceptive accuracy, indicating that participants with higher accuracy in heartbeat counting demonstrated greater HRV.

The characteristics of the studies that addressed interoception are summarized in the Supplementary Table S4.

#### 3.2.2 Stress

Of the 18 studies included in the scoping review, 11 dealt with stress: 8 focused on chronic stress (75–79, 82, 84, 85), and 3 on stress reactivity (80, 81, 83).

##### 3.2.2.1 Chronic stress

Studies addressed chronic stress in a variety of ways. Magal et al. (75) described chronic stress as a process in which external or internal demands (stressors) exceed personal resources for a prolonged period of time. They classified participants into high- and low-stress categories, differentiating stress from various sources (e.g., social, work) and quantified it with the Trier Inventory for Chronic Stress questionnaire. Furthermore, they used a Charge 3 (Fitbit) to collect physical and behavioural measures such as HR, as well as data related to sleep and activity, with the aim of predicting chronic stress. Tsujikawa et al. (76) created an accurate chronic stress estimation system. They used an Embrace2 (Empatica) wrist-worn during work hours on weekdays to collect EDA and ACC (accelerometry) measures. Also, they utilized the Big Five Personality Traits questionnaire to classify users, and the Perceived Stress Scale questionnaire (PSS) as ground truth. Hirten et al. (77) proposed an evaluation of the perceived and physiological consequences of stressors to identify individuals at risk of chronic stress. To this end they proposed HRV as a marker of physiological stress on the autonomic nervous system and utilized the Apple Watch PPG sensor to measure it. Perceived stress was measured using the PSS-4 at baseline and through EMA. Nakashima et al. (82) aimed to improve the early recognition of chronic stress by more effectively monitoring physiological signals in people's daily lives. They use EDA as a physiological signal of stress and the PSS-10 to determine one-month accumulated chronic stress as the ground truth. Physiological data were collected using an Embrace2 (Empatica) wrist-worn device. Berrocal et al. (85), proposed to use peers and subjective data from participants, along with physiological measures, to define a model that predicts individuals' self-assessment of stress. They used HRV obtained with an E4 (Empatica) wristband for physiological data, while self-assessments were conducted through EMA. The model also incorporated variables related to smartphone usage.

Rodrigues et al. (78) proposed combining physiological and self-report data to quantify chronic stress and examined variations in these measures between work shifts and off-shifts. For physiological metrics, they used an ECG patch (electrocardiogram) to extract various HRV indices. For self-reported measures, the PSS was employed to assess chronic stress. Van Kraaij et al. (79) analyzed the long-term HR response to chronic stress, accounting for potential gender and age biases. They defined chronic stress as long-term perceived stress, measured using the PSS-10. Integrating methods and findings from previous research, they evaluated the HR response as a physiological marker of chronic stress using a Gobio ECG chest patch for HR measurements. In a separate study protocol, Wilbur et al. (84) aimed to evaluate the feasibility of using biometric data and salivary cortisol, along with qualitative data, to evaluate occupational stress in fishermen and its potential short- and long-term health impacts. Biometric data were recorded using a wearable garment with embedded textile sensors (ECG, respiratory inductance plethysmography, and ACC). This study proposed HRV, respiration, and movement metrics as proxies for physical and psychological stress.

The studies used various low-level features in their models. Among the studies that obtained HR measures, two conducted circadian analyses of HR (a group of techniques used to study 24-hour biological rhythms and their variability) (79, 84), and one study (75) performed a cosinor analysis of HR (a method that fits a cosine curve to time-series data to identify rhythmic patterns), in combination with other statistical HR parameters. Several studies (77, 78, 84, 85) utilized various HRV indices, such as RMSSD, the average value of NN interval (AVNN), percentage of successive NN intervals that differ by more than 50 ms (pNN50) and the ratio of LF (low frequency) and HF (high frequency) power bands (LF/HF) (78), or the standard deviation of NN intervals (SDNN) (77). Furthermore, the study (77) employed a cosinor analysis of HRV. Two studies incorporated EDA measures. The first study (82) used statistical values (average, median, standard deviation) and power spectral density bins (which represent the amount of signal power within specific frequency ranges, used to detect patterns or variability in time-series data), while the other (76) analyzed EDA features in the frequency domain. Only one study examined respiratory features (84), specifically breathing rate.

Among the studies that included behavioural measures, one study (75) used sleep features (sleep onset and sleep regularity, among others). Two studies utilized activity features, such as metrics related to steps and time spent at different activity levels (75), or a physical activity index (84). Additionally, another study (85) proposed features derived from smartphone usage.

It is important to note that data obtained from ACC are utilized in some studies to account for the influences of physical activity on physiological signals (76, 79, 82, 83).

In addition to data collected from devices, many of the studies incorporated demographic information in their analyses of stress, such as age and sex. Furthermore, one study used observational data recorded by the principal investigator, who noted key events involving participants (84). This study also included measurements of salivary cortisol. In one study protocol (85) researchers propose something similar: peers (defined as close friends or family members) were employed as observers for the study participants, providing observer-reported outcomes regarding participant stress levels. Magal et al. (75) integrated participant's smoking status and the Normalized Difference Vegetation Index (*N*DVI) in their model. Lastly, Hirten et al. (77) examined features such as the number of COVID-19 cases in the community, and surveys assessing emotional support, resilience, optimism, and quality of life to model longitudinal changes in stress.

##### 3.2.2.2 Stress reactivity

With respect to the studies on stress reactivity, the first one (80) operationalized this construct based on a model from previous research and used HRV as an indicator of physiological stress reactivity and recovery in real-life scenarios. Physiological data was obtained using an ECG patch, and chronic stress levels were assessed at baseline through questionnaires, including the Effort-Reward Imbalance scale and the Job Demand and Control scale. In another study, stress reactivity was explored in the context of early life stress (81). EDA reactivity was used as a measure of physiological stress reactivity, evaluated with a Q-sensor (Affectiva) wrist monitor. EMA data was collected through a smartphone provided to participants and EDA data were retrieved upon return of the device. The last study examined the use of wearable technology to identify digital phenotypes associated with stress in daily life (83). The authors introduced a measure called the dynamic range of physiological features as a proxy for physiological stress reactivity, calculated as the average difference per physiological feature between low and high stress states. This study used two types of wearable devices: a chest patch measuring ECG and ACC, and a wrist-worn device measuring skin conductance, skin temperature, and ACC. In addition, a smartphone was used to collect contextual data, including location and audio features.

Studies used a variety of features in their models. Smets et al. (83) calculated 18 physiological features specifically 6 ECG features, 8 skin conductance features, and 4 skin temperature features. Schilling et al. (80) used HRV, in particular RMSSD, as a measure of parasympathetic activity (used as an indicator of physiological stress reactivity and recovery in real-life measurements). Last, in the study by Timmons et al. (81) EDA was quantified as the skin conductance level measured in micro siemens.

##### 3.2.2.3 Stress studies data processing

Regarding data processing, among the 11 studies that addressed stress, 5 employed ML techniques for classification (type 1 studies). One study developed a custom classification system based on correlation maximization, which outperformed k-means clustering (69.1% vs. 56.8% accuracy) in estimating changes in PSS scores using EDA and ACC data  (76). Another employed random forest models using self-perceived stress based on PSS as ground truth, reporting modest overall performance (F1-score = 0.43), although physiological responses to stress varied substantially across individuals  (83). A third study applied support vector regression to compare two representations of physical activity, activity state and activity magnitude, for predicting chronic stress, finding that the former significantly outperformed the latter (MAE: 3.34 vs. 4.46), though classification accuracy was not reported (82) . Another study used a support vector machine classifier to detect chronic stress related to social tension, based on the Trier Inventory for Chronic Stress and derived features from HR, sleep, activity, and smoking status, achieving 79% classification accuracy  (75). The final study employed a ML classification model (unspecified) but did not report results, as it was an ongoing project  (85). The last three studies also incorporated statistical analysis methods (type 2 studies). Across these studies, features derived from HRV, EDA, HR, physical activity and sleep appeared relevant for chronic stress classification or prediction.

Among the studies that used statistical methods (type 2 studies) and reported results, one (78) employed nonparametric tests (Wilcoxon Signed test and Kruskal–Wallis test) and found that AVNN and LF/HF ratios derived from HRV measurements had strong discriminatory power for short-term stress classification. Another study (79) used a mixed design model and reported a significant interaction between HR, chronic stress, gender, and HR circadian rhythm. A third study (80) applied multilevel modelling and regression analysis, finding inconsistent results overall, but suggesting that individuals with higher cardiorespiratory fitness exhibited reduced physiological stress reactivity (as indexed by HRV) in response to acute work stress.

The characteristics of the studies that addressed stress are summarized in the Supplementary Table S5.

#### 3.2.3 Self-regulation

Of the 18 studies included in the scoping review, 5 included measures of self-regulation. Among these, one study (90) addressed three constructs related to self-regulation (regulation of emotion, cognition, and self-reflection) and four studies addressed emotion regulation (ER) (86–89).

The four studies that included measures of ER explored its relationship with other mental states or disorders including sleep bruxism (86), students' learning performance (87), mindfulness and well-being (88), and emotional eating (89). The four studies proposed measures of ER based on previous research. They used wearable devices to obtain physiological data, with 2 studies using ECG chest patches and 2 studies utilizing E4 (Empatica) wristband devices. Only one study (86) relied on self-reports for subjective measures of ER. Notably, all four studies used HRV as a proxy for ER. The study that addressed self-regulation aimed to identify assays at different levels of measure and assess their predictive value and impact on the adherence, mood and weight outcomes of the intervention (90).

In a study protocol, Kreibig et al. (86) aimed to assess the use and effectiveness of ER on both trait and state timescales. They employed HRV obtained from an ECG patch as a physiological measure of ER, whereas subjective assessments comprised questionnaires provided at baseline and daily life via EMA for real-time reporting of ER strategies. Another study used HRV as a physiological marker of regulatory resources; specifically, they used RMSSD, a time-domain feature, to analyze state vagal activity (88). A different study employed HRV as a transdiagnostic biosignal of ER, incorporating features in the time and frequency domains (89). Lastly, a study simply stated that ER was directly calculated from HRV (87). Interestingly, the 4 studies that address ER are of type 3 (the studies did not involve predicting ER or searching for new ER biomarkers).

The study of Williams et al. (90) collected assays of 3 self-regulation targets (emotion, cognition, and self-reflection) in multiple settings. For naturalistic assays, the study used participants' smartphones on which the Mindstrong application was installed. This allowed the collection of 288 phone-use feature variables and GPS data related to self-regulation (emotional states, behavioural changes and cognitive regulation) throughout the study period. Functional imaging of large-scale brain circuits, specifically the “Affective,” “Cognitive Control,” and “Default Mode” networks, was chosen as the gold standard measures. The study intends to use unsupervised and supervised data analysis models to establish the relationship between the assays and the settings. This paper was a study protocol and did not present results.

The characteristics of the studies that addressed self-regulation are summarized in the Supplementary Table S6.

## 4 Discussion

### 4.1 Principal findings

In this scoping review, our aim was to synthesize the digital phenotyping studies that measure and predict interoception, chronic stress, and self-regulation in adults.

First, it is important to note that relatively few studies were identified: 11 focused on chronic stress and stress reactivity, 5 on self-regulation, and 2 on interoception. The limited number of studies identified makes it challenging to draw definitive conclusions, however, the findings can provide valuable insights into current research trends and highlight significant gaps in the literature.

The domain of stress research is well-established, particularly regarding the physiological mechanisms underlying the stress response. Acute stress is commonly studied in laboratory settings using standardized tasks designed to elicit stress (93–95). While longitudinal studies have explored acute stress in daily life (64, 96) there is a significant gap in research focusing on pathological stress, including chronic stress and allostatic load, in real-world settings. These forms of stress, characterized by prolonged dysregulation of stress responses, remain underexplored in ecologically valid longitudinal studies despite their profound implications for physical and mental health. The findings of this review, which analyzed 11 stress-related studies, 8 addressing chronic stress and 3 focusing on stress reactivity, highlight the limited research available on this topic.

Self-regulation and interoception are broad domains and present additional challenges for operationalization and measurement, even more through digital phenotyping. Self-regulation studies included in this review focus primarily on ER. The only study that addresses self-regulation in a comprehensive manner is an interesting study protocol (90) aimed at exploring self-regulation mechanisms related to behavior change, specifically to improve mood and weight. Aside from this study, which seeks relationships between variables and aims to predict outcomes, the four studies on ER rely on findings from previous research to operationalize ER and do not pursue new digital biomarkers related to ER mechanisms.

Interoception is a complex domain that is still not fully understood, involving multiple dimensions related to the perception and processing of internal bodily signals. Traditionally, interoception has been assessed through behavioral accuracy tests, self-report measures, and the analysis of neural signals associated with interoceptive processing. The two studies included in this review focused on cardiac interoception, both use a smartphone to perform interoceptive accuracy tasks and both obtain HRV metrics that attempt to correlate with various interoceptive dimensions. While these studies meet the inclusion criteria of the review, they slightly deviate from the continuous longitudinal data collection model central to digital phenotyping. However, we included them because they may serve as useful models for future studies that aim to identify digital biomarkers relevant to interoception, despite their reliance on shorter-term data collection paradigms.

This review focused on studies using smartphones and commercially available wearable devices that can be easily integrated into daily life. For physiological measurements, the devices used most frequently in the studies were ECG chest patches and wrist-worn wearables. The frequent use of ECG patches can be attributed to the focus of most studies on assessing HR and HRV. Other studies relied on PPG wrist-worn sensors to capture these metrics. ECG patches were commonly used in shorter studies (averaging 6 days in this review), probably due to the relative discomfort they pose compared to wrist-worn wearables. As PPG sensors continue to improve in accuracy and offer relative comparability to ECGs, as demonstrated in recent validation studies (97–100), they appear to be more suitable for longitudinal studies that require continuous data collection in daily life. However, ambulatory PPG recordings are subject to limitations, including sensitivity to motion artifacts, posture and activity confounders, and variability across devices and individuals (101, 102). In addition to ECG and PPG sensors, EDA sensors were employed in 4 studies, while ACC sensors were used in 3 (although 4 other studies incorporated ACC data to account for the effects of physical activity on physiological signals). One study utilized a smartwatch, and another employed a sensorized shirt.

Beyond the studies included in this review, related literature indicates that the use of commercially available wrist-worn wearables and smartphones (e.g., Fitbit, Apple Watch, Empatica) is widespread in longitudinal studies addressing psychological and neurological conditions (such as depression, schizophrenia or Parkinson Disease) due to their accessibility and integration into daily routines (52, 103–107). Additional wearable device types used in related studies include rings, earphones, and e-textiles equipped with sensors (108, 109), which expand the range of physiological and behavioral data that can be captured unobtrusively in real-world settings. Furthermore, EEG wearable devices (110) have emerged as promising tools for monitoring neural activity in naturalistic contexts, offering new opportunities for brain health research and the study of neural dynamics associated with stress, self-regulation and interoception. These devices, however, face practical and technical limitations, including battery life, user comfort in long-term wear, inconsistent data transmission and storage, technical differences across devices and platforms, or security and privacy considerations (109, 111), which need to be considered when interpreting findings.

Among the studies included in this review, the use of smartphones for behavioral tracking was limited, with only 3 studies that used smartphones for this purpose. Notably, the two studies that examined interoception utilized smartphones to collect physiological data by detecting heartbeats through smartphone cameras using PPG. However, smartphones were more commonly used for EMA, with 8 studies employing them for active data collection. EMA has been incorporated into the proposed digital phenotyping model (Figure 1) as an essential component. It involves capturing self-reported data on subjective experiences, emotions, and perceptions in real time contexts. EMA is essential for complementing objective sensor data, offering valuable insights into an individual's lived experiences, and deepening the understanding of the interplay between subjective and objective markers of health and behavior.

The limited use of smartphones for behavioral tracking in this review contrasts with its extensive application in studies focused on other mental states or diseases, such as depression or schizophrenia, where smartphone-based measures are often central to data collection (45, 49, 109, 112). This suggests that there may be an underutilized potential for smartphone data in the domains reviewed here, especially given the advances in mobile sensing technology. Since most adults in developing countries now have and use a smartphone, its use facilitates conducting research in real world settings, leveraging everyday environments. The data generated by these increasingly sophisticated smartphone sensors and smartphone use patterns seems ideal for capturing various social and behavioural dimensions of psychiatric and neurological diseases.

Some studies included in the review used raw sensor data (minimally processed data obtained directly from the sensors), while others relied on data processed by the device software [such as data provided by Fitbit in (75)]. Using raw data requires thorough signal analysis and preprocessing, including filtering, interpolation, and artifact removal. The choice between raw and processed data depends on the researchers' goals and study design. Some argue that consumer-grade wearable devices and smartphone apps are not intended for biomedical research, as they do not produce research-grade data (113). However, others have examined the validity of data provided by these devices and found that they are in good agreement with gold standard measurements (100, 114, 115).

Many digital phenotyping studies rely on multiple sensing modalities. Representing and integrating these diverse streams poses significant analytical challenges, and various structured frameworks exist to guide this process (116, 117). The central integration step is multimodal data fusion, which combines information from various modalities into a unified representation for subsequent analysis or modeling. Fusion techniques are typically categorized by the integration stage: early (feature-level), intermediate (representation-level), and late (decision-level) (116, 117). Approaches and reporting practices differ substantially across studies, limiting transparency. Critically, in the studies included in this review, none explicitly refer to “data fusion” or “multimodal fusion” using formal terminology. However, all studies employing multiple modalities, when methodology was reported, integration was consistently performed at the feature level (early fusion).

The most used measure in the studies was HRV, which was employed in 12 studies. Of these, 4 studies on emotion regulation used HRV as a physiological marker of regulatory resources, 2 studies on interoception utilized it to correlate with measures of interoceptive accuracy and interoceptive sensibility, and 6 studies on stress used HRV as an index of autonomic nervous system functioning. These findings were anticipated, as extensive research has shown a correlation between poor mental health and impaired HRV (63, 118–123). Stress-related studies included in this review also employed EDA features as indicators of sympathetic nervous system activity, as well as measures of HR, ACC, and temperature, all of which are associated with the stress response (62, 95).

The findings related to the domains of interest across studies are varied. The two studies on interoception explore the relationship between HRV and different interoceptive dimensions, such as interoceptive accuracy and interoceptive sensibility, with contradictory results. Study (92) does not report any significant relationship, while study (91) finds a positive association between HRV and interoceptive accuracy. The results of the latter study align with recent laboratory-based research that has identified a relationship between HRV and interoceptive accuracy (41, 124), suggesting that HRV may be a potential physiological digital biomarker for certain interoceptive processes, but there are still limited studies to consider these data conclusive.

Studies addressing stress used either statistical or machine learning methods to examine associations or classify stress states based on physiological and behavioral features. Among the 11 stress-related studies, 5 applied machine learning techniques. Reported performance varied, with classification accuracy ranging from 56.8% to 79%, and one study reporting an F1-score of 0.43. The studies employed a variety of models, including random forest, support vector machines, and regression-based approaches, although methodological details were often limited or insufficiently reported. Sample sizes and monitoring durations were variable and generally modest. Despite this heterogeneity, features derived from HRV, EDA, HR, physical activity and sleep appeared relevant for chronic stress classification or prediction. The study achieving the highest accuracy (79%) also incorporated sociodemographic and lifestyle data, suggesting that integrating such contextual variables may enhance predictive performance. On the other hand, six studies relied on statistical methods to explore associations between sensor-derived data and stress. These analyses underscored the capacity of HRV features to differentiate stress levels and revealed consistent associations with a range of physiological, behavioral, contextual, and self-reported indicators, including questionnaire-based measures of chronic stress and stress reactivity. Moderating factors such as gender, circadian rhythm, and cardiorespiratory fitness were also identified. Together, these findings support existing evidence from laboratory studies (93–95) and underscore the multifactorial nature of stress responses, emphasizing the need to integrate physiological, behavioral, and contextual data in future research.

Beyond stress, the methodological patterns observed across the included studies suggest a gradual shift in the field: while traditional statistical modeling remains the dominant analytical approach, there is growing adoption of machine learning methods, particularly for predictive tasks. This evolution reflects broader trends in digital health research, although the limited number of studies and the heterogeneity of methods should be taken into account.

Considering these methodological aspects, it is also important to acknowledge that, even though large-scale digital phenotyping initiatives exist, the evidence base for these three domains remains limited. In our scoping review, the largest study included 1,002 participants, whereas most studies had substantially smaller samples. By contrast, several large-scale projects in related fields demonstrate the feasibility of enrolling thousands of participants: Zhang et al. (125) with *n* = 10,129 UK participants used Fitbit wearables to identify and predict depression and anxiety; Dai et al. (126) analyzed a public dataset of *n* = 8,996 individuals to detect depression and anxiety with activity trackers; and Wyatt et al. (127) analyzed data from *n* = 208,818 activity tracker users across 34 countries to examine associations between COVID-19 and physical activity, HR, and sleep. This comparison illustrates the feasibility of conducting population-scale studies in related domains.

### 4.2 Current limitations and future directions

Beyond these study-specific findings, a broader interpretation of the field reveals important limitations and opportunities that extend across interoception, chronic stress, and self-regulation research. At present, digital phenotyping in these domains is constrained by the very small number of available studies, the predominance of short monitoring periods, and the scarcity of ecologically valid, long-term datasets. Research on pathological stress, particularly chronic stress and allostatic load, also remains limited in real-world settings despite its clinical relevance. Digital phenotyping approaches to self-regulation and interoception are even more underdeveloped: operational definitions are heterogeneous, core constructs are difficult to capture through passive sensing, and existing studies focus on narrow aspects such as emotion regulation or cardiac interoception, leaving broader dimensions unexplored.

Methodologically, the field continues to rely heavily on ECG chest patches and standard wrist-worn wearables, with limited adoption of alternative sensors, new device formats, or multimodal measurement strategies that could expand the scope of detectable signals. Smartphone-based behavioral sensing is similarly underused in these domains. Reporting practices are also inconsistent: preprocessing pipelines, feature extraction procedures, and validation strategies are often insufficiently detailed, complicating reproducibility and cross-study comparability. Although machine-learning approaches are becoming more common, they remain underexploited, and when applied, performance tends to be modest due to small samples, restricted sensor diversity, and short monitoring durations.

These limitations open clear avenues for future research. Population-based, long-term studies are needed to establish robust digital markers of chronic stress, self-regulation, and interoception. Advancing these domains will require identifying which theoretical constructs are most amenable to digital measurement (e.g., beyond emotion regulation within self-regulation, or beyond cardiac accuracy within interoception), broadening sensing modalities, and validating the use of commercial wearables and smartphones at scale. The integration of richer behavioral, contextual, and environmental data represents another promising direction to improve predictive performance. Finally, clearer methodological reporting and more rigorous, well-powered machine-learning pipelines will be essential to consolidate progress and ensure replicability. Collectively, these future efforts have the potential to transform digital phenotyping into a scalable, multimodal framework capable of capturing complex psychophysiological processes in everyday life.

### 4.3 Limitations of the review methodology

This scoping review has limitations that should be considered in future research.

First, we reviewed only published studies, excluding materials such as research theses or government reports. Consequently, future work could expand this scoping review by incorporating gray literature to provide a more comprehensive overview of the research landscape. Another potential limitation of this study arises from the search terms used to identify wearable devices, as there is a wide variety of wearables and variable terminology to describe them. This variability may have resulted in the omission of relevant devices or studies. Expanding and refining the search terms to encompass a broader range of terminologies and device categories would help address this limitation in future reviews. And finally, given the broad scope of the domains explored in this review, some studies may focus on specific constructs rather than addressing the overarching domain, leading to their exclusion from the search results. This challenge is made more difficult by the lack of consensus in defining many of these constructs and domains. Although challenging, this could be mitigated by conducting more focused reviews on individual domains and refining search terms accordingly.

Addressing these limitations would not only improve the comprehensiveness of future reviews but also help standardize terminologies and methodologies in the field.

## 5 Conclusions

This scoping review reveals that, although research on digital phenotyping for interoception, chronic stress, and self-regulation is still in its early stages, existing studies already demonstrate the feasibility of capturing meaningful physiological and behavioral signals using wearable devices and smartphones. Key patterns emerge: heart rate variability is consistently employed across domains; smartphones remain largely underutilized for behavioral tracking; and most studies rely on short-term monitoring with modest sample sizes. These findings highlight both the current limitations and the tremendous potential to advance brain and mental health research through scalable digital phenotyping approaches. Future research should prioritize long-term, multimodal data collection in larger and more diverse populations; integrate novel devices, sensors, and smartphone-derived behavioral measures; refine and expand the operationalization of key constructs; and ensure transparent rigorous methodological reporting. Developing and applying advanced machine learning and data-analysis strategies will be crucial to establish robust and reliable digital biomarkers capable of capturing complex psychophysiological processes.

By addressing these challenges, digital phenotyping has the potential to capture proximal indicators of mechanisms proposed to underline behavior change, specifically interoception, chronic stress, and self-regulation, and to deepen our understanding of how these processes unfold in daily life, ultimately driving progress and supporting brain and mental health research.

## Data Availability

The original contributions presented in the study are included in the article/Supplementary Material, further inquiries can be directed to the corresponding author.

## References

[1] World Health Organization. Over 1 in 3 people affected by neurological conditions, the leading cause of illness and disability worldwide (2024). (Accessed May 27, 2024).

[2] GBD. 2021 Nervous system disorders collaborators. Global, regional, and national burden of disorders affecting the nervous system, 1990–2021: a systematic analysis for the global burden of disease study 2021. Lancet Neurol. (2024) 23(4):344–81. 10.1016/S1474-4422(24)00038-3 38493795 PMC10949203

[3] Livingston G Huntley J Sommerlad A Ames D Ballard C Banerjee S Dementia prevention, intervention, and care: 2020 report of the lancet commission. Lancet. (2020) 396(10248):413–46. 10.1016/S0140-6736(20)30367-6 32738937 PMC7392084

[4] Sumner JA Carey RN Michie S Johnston M Edmondson D Davidson KW. Using rigorous methods to advance behaviour change science. Nat Hum Behav. (2018) 2(11):797–9. 10.1038/s41562-018-0471-8 30931398 PMC6437667

[5] Kupst MJ Butt Z Stoney CM Griffith JW Salsman JM Folkman S Assessing stress and self-efficacy for the NIH toolbox for neurological and behavioral function. Anxiety Stress Coping. (2015) 28(5):531. 10.1080/10615806.2014.994204 25577948 PMC4515370

[6] Smyth JM Zawadzki MJ Marcusson-Clavertz D Scott SB Johnson JA Kim J Computing components of everyday stress responses: exploring conceptual challenges and new opportunities. Perspect Psychol Sci. (2023) 18(1):110–24. 10.1177/17456916221082108 35904963 PMC9851922

[7] McEwen BS. What is the confusion with cortisol? Chronic Stress (Thousand Oaks). (2019) 3:2470547019833647. 10.1177/2470547019833647 31608312 PMC6788742

[8] Khalsa SS Adolphs R Cameron OG Critchley HD Davenport PW Feinstein JS Interoception and mental health: a roadmap. Biol Psychiatry Cogn Neurosci Neuroimaging. (2018) 3(6):501–13. 10.1016/j.bpsc.2017.12.004 29884281 PMC6054486

[9] Brewer R Murphy J Bird G. Atypical interoception as a common risk factor for psychopathology: a review. Neurosci Biobehav Rev. (2021) 130:470–508. 10.1016/j.neubiorev.2021.07.036 34358578 PMC8522807

[10] Zhou H Zou H Dai Z Zhao S Hua L Xia Y Interoception dysfunction contributes to the negative emotional bias in Major depressive disorder. Front Psychiatry. (2022) 13:874859. 10.3389/fpsyt.2022.874859 35479498 PMC9035634

[11] Kwasnicka D Dombrowski SU White M Sniehotta F. Theoretical explanations for maintenance of behaviour change: a systematic review of behaviour theories. Health Psychol Rev. (2016) 10(3):277–96. 10.1080/17437199.2016.1151372 26854092 PMC4975085

[12] Hennessy EA Johnson BT Acabchuk RL McCloskey K Stewart-James J. Self-Regulation mechanisms in health behaviour change: a systematic meta-review of meta-analyses, 2006–2017. Health Psychol Rev. (2020) 14(1):6. 10.1080/17437199.2019.1679654 31662031 PMC7571594

[13] Smyth JM Sliwinski MJ Zawadzki MJ Scott SB Conroy DE Lanza ST Everyday stress response targets in the science of behavior change. Behav Res Ther. (2018) 101:20–9. 10.1016/j.brat.2017.09.009 29031538 PMC5801200

[14] Miller AL Gearhardt AN Fredericks EM Katz B Shapiro LF Holden K Targeting self-regulation to promote health behaviors in children. Behav Res Ther. (2018) 101:71–81. 10.1016/j.brat.2017.09.008 29050636 PMC5801044

[15] Nielsen L Riddle M King JW Aklin WM Chen W Clark D The NIH science of behavior change program: transforming the science through a focus on mechanisms of change. Behav Res Ther. (2018) 101:3–11. 10.1016/j.brat.2017.07.002 29110885 PMC5756516

[16] Plaitano EG McNeish D Bartels SM Bell K Dallery J Grabinski M Adherence to a digital therapeutic mediates the relationship between momentary self-regulation and health risk behaviors. Front Digit Health. (2025) 7:1457772. 10.3389/fdgth.2025.1467772 PMC11841403 39981105

[17] Scherer EA Metcalf SA Whicker CL Bartels SM Grabinski M Kim SJ Momentary influences on self-regulation in two populations with health risk behaviors: adults who smoke and adults who are overweight and have binge-eating disorder. Front Digit Health. (2022) 4:798895. 10.3389/fdgth.2022.798895 35373179 PMC8971561

[18] Lazarus RS Folkman S. Stress, Appraisal, and Coping. New York: Springer Publishing Company (1984).

[19] McEwen BS. The brain on stress: toward an integrative approach to brain, body and behavior. Perspect Psychol Sci. (2013) 8(6):673. 10.1177/1745691613506907 25221612 PMC4159187

[20] Thayer JF Mather M Koenig J. Stress and aging: a neurovisceral integration perspective. Psychophysiology. (2021) 58(7):e13804. 10.1111/psyp.13804 33723899

[21] O'Connor DB Thayer JF Vedhara K. Stress and health: a review of psychobiological processes. Annu Rev Psychol. (2021) 72(1):663–88. 10.1146/annurev-psych-062520-122331 32886587

[22] Fava GA McEwen BS Guidi J Gostoli S Offidani E Sonino N. Clinical characterization of allostatic overload. Psychoneuroendocrinology. (2019) 108:94–101. 10.1016/j.psyneuen.2019.05.028 31252304

[23] Lupien SJ Juster R Raymond C Marin M. The effects of chronic stress on the human brain: from neurotoxicity, to vulnerability, to opportunity—scienceDirect. Front Neuroendocrinol. (2018) 49:91–105. 10.1016/j.yfrne.2018.02.001 29421159

[24] McEwen BS Stellar E. Stress and the individual: mechanisms leading to disease. Arch Intern Med. (1993) 153(18):2093–101. 10.1001/archinte.1993.00410180039004 8379800

[25] Bisht K Sharma K Tremblay M. Chronic stress as a risk factor for Alzheimer’s disease: roles of microglia-mediated synaptic remodeling, inflammation, and oxidative stress. Neurobiol Stress. (2018) 9:9. 10.1016/j.ynstr.2018.05.003 29992181 PMC6035903

[26] McEwen BS Akil H. Revisiting the stress concept: implications for affective disorders. J Neurosci. (2020) 40(1):12–21. 10.1523/JNEUROSCI.0733-19.2019 31896560 PMC6939488

[27] Guidi J Lucente M Sonino N Fava G. Allostatic load and its impact on health: a systematic review. Psychother Psychosom. (2020) 90(1):11–27. 10.1159/000510696 32799204

[28] Parker HW Abreu AM Sullivan MC Vadiveloo MK. Allostatic load and mortality: a systematic review and meta-analysis. Am J Prev Med. (2022) 63(1):131–40. 10.1016/j.amepre.2022.02.003 35393143

[29] McEwen BS. Stress, adaptation, and disease: allostasis and allostatic load. Ann N Y Acad Sci. (1998) 840(1):33–44. 10.1111/j.1749-6632.1998.tb09546.x 9629234

[30] Turner AI Smyth N Hall SJ Torres SJ Hussein M Jayasinghe SU Psychological stress reactivity and future health and disease outcomes: a systematic review of prospective evidence. Psychoneuroendocrinology. (2020) 114:104599. 10.1016/j.psyneuen.2020.104599 32045797

[31] Carroll D Ginty AT Whittaker AC Lovallo WR Rooij S. The behavioural, cognitive, and neural corollaries of blunted cardiovascular and cortisol reactions to acute psychological stress. Neurosci Biobehav Rev. (2017) 77:74. 10.1016/j.neubiorev.2017.02.025 28254428 PMC6741350

[32] Agorastos A Chrousos GP. The neuroendocrinology of stress: the stress-related continuum of chronic disease development. Mol Psychiatry. (2022) 27(1):502–13. 10.1038/s41380-021-01224-9 34290370

[33] Eisenberg IW Bissett PG Canning JR Dallery J Enkavi AZ Whitfield-Gabrieli S Applying novel technologies and methods to inform the ontology of self-regulation. Behav Res Ther. (2018) 101:46–57. 10.1016/j.brat.2017.09.014 29066077 PMC5801197

[34] Romer AL Hariri AR Strauman TJ. Regulatory focus and the *p* factor: evidence for self-regulatory dysfunction as a transdiagnostic feature of general psychopathology. J Psychiatr Res. (2021) 137:178–85. 10.1016/j.jpsychires.2021.02.051 33684642 PMC8085096

[35] Strauman TJ. Self-Regulation and psychopathology: toward an integrative translational research paradigm. Annu Rev Clin Psychol. (2017) 13:497–523. 10.1146/annurev-clinpsy-032816-045012 28375727

[36] Kelley WM Wagner DD Heatherton TF. In search of a human self-regulation system. Annu Rev Neurosci. (2015) 38:389–411. 10.1146/annurev-neuro-071013-014243 25938728 PMC4530781

[37] Murphy J Brewer R Catmur C Bird G. Interoception and psychopathology: a developmental neuroscience perspective. Dev Cogn Neurosci. (2016) 23:45–56. 10.1016/j.dcn.2016.12.006 28081519 PMC6987654

[38] Price CJ Hooven C. Interoceptive awareness skills for emotion regulation: theory and approach of mindful awareness in body-oriented therapy (MABT). Front Psychol. (2018) 9:798. 10.3389/fpsyg.2018.00798 29892247 PMC5985305

[39] Petzschner FH Garfinkel SN Paulus MP Koch C Khalsa SS. Computational models of interoception and body regulation. Trends Neurosci. (2021) 44(1):63–76. 10.1016/j.tins.2020.09.012 33378658 PMC8109616

[40] Smith R Thayer JF Khalsa SS Lane RD. The hierarchical basis of neurovisceral integration. Neurosci Biobehav Rev. (2017) 75:274–96. 10.1016/j.neubiorev.2017.02.003 28188890

[41] Pinna T Edwards DJ. A systematic review of associations between interoception, vagal tone, and emotional regulation: potential applications for mental health, wellbeing, psychological flexibility, and chronic conditions. Front Psychol. (2020) 11:1792. 10.3389/fpsyg.2020.01792 32849058 PMC7419655

[42] Pace-Schott EF Amole MC Aue T Balconi M Bylsma LM Critchley H Physiological feelings. Neurosci Biobehav Rev. (2019) 103:267–304. 10.1016/j.neubiorev.2019.05.002 31125635

[43] Cornelius T Derby L Connell Bohlen L Birk JL Rothman AJ Johnston M Linking measures to mechanisms of action: an expert opinion study. Br J Health Psychol. (2023) 28(1):98–115. 10.1111/bjhp.12614 35781731 PMC9807686

[44] Torous J Onnela J Keshavan M. New dimensions and new tools to realize the potential of RDoC: digital phenotyping via smartphones and connected devices. Transl Psychiatry. (2017) 7(3):e1053. 10.1038/tp.2017.25 28267146 PMC5416670

[45] Huckvale K Venkatesh S Christensen H. Toward clinical digital phenotyping: a timely opportunity to consider purpose, quality, and safety. NPJ Digit Med. (2019) 2:88. 10.1038/s41746-019-0166-1 31508498 PMC6731256

[46] Dlima SD Shevade S Menezes SR Ganju A. Digital phenotyping in health using machine learning approaches: scoping review. JMIR Bioinform Biotechnol. (2022) 3(1):e39618. 10.2196/39618 38935947 PMC11135220

[47] Lee K Lee TC Yefimova M Kumar S Puga F Azuero A Using digital phenotyping to understand health-related outcomes: a scoping review. International journal of medical informatics (Shannon. Ireland. (2023) 174:105061. 10.1016/j.ijmedinf.2023.105061 37030145

[48] Trifan A Oliveira M Oliveira JL. Passive sensing of health outcomes through smartphones: systematic review of current solutions and possible limitations. JMIR Mhealth Uhealth. (2019) 7(8):e12649. 10.2196/12649 31444874 PMC6729117

[49] Cornet VP Holden RJ. Systematic review of smartphone-based passive sensing for health and wellbeing. J Biomed Inform. (2018) 77:120–32. 10.1016/j.jbi.2017.12.008 29248628 PMC5793918

[50] Ettore E Müller P Hinze J Riemenschneider M Benoit M Giordana B Digital phenotyping for differential diagnosis of Major depressive episode: narrative review. JMIR Ment Health. (2023) 10(1):e37225. 10.2196/37225 36689265 PMC9903183

[51] Miller ML Raugh IM Strauss GP Harvey PD. Remote digital phenotyping in serious mental illness: focus on negative symptoms, mood symptoms, and self-awareness. Biomarkers Neuropsychiatry. (2022) 6:100047. 10.1016/j.bionps.2022.100047

[52] Kourtis LC Regele OB Wright JM Jones GB. Digital biomarkers for Alzheimer’s disease: the mobile/wearable devices opportunity. NPJ Digit Med. (2019) 2:9. 10.1038/s41746-019-0084-2 31119198 PMC6526279

[53] Lott SA Streel E Bachman SL Bode K Dyer J Fitzer-Attas C Digital health technologies for Alzheimer’s disease and related dementias: initial results from a landscape analysis and community collaborative effort. J Prev Alzheimer's Dis. (2024) 11(5):1480–9. 10.14283/jpad.2024.103 39350395 PMC11436391

[54] Babrak LM Menetski J Rebhan M Nisato G Zinggeler M Brasier N Traditional and digital biomarkers: two worlds apart? Digit Biomark. (2019) 3(2):92–102. 10.1159/000502000 32095769 PMC7015353

[55] Kyriazakos S Pnevmatikakis A Cesario A Kostopoulou K Boldrini L Valentini V Discovering composite lifestyle biomarkers with artificial intelligence from clinical studies to enable smart eHealth and digital therapeutic services. Front Digit Health. (2021) 3:648190. 10.3389/fdgth.2021.648190 34713118 PMC8521973

[56] Nguyen B Ivanov M Bhat V Krishnan S. Digital phenotyping for classification of anxiety severity during COVID-19. Front Digit Health. (2022) 4:877762. 10.3389/fdgth.2022.877762 36310921 PMC9612961

[57] Boukhechba M Baglione AN Barnes LE. Leveraging Mobile sensing and machine learning for personalized mental health care. Ergon Des. (2020) 28(4):18–23. 10.1177/1064804620920494

[58] Yin H Zhu H Gu J Qin H Ding W Guo N Mobile-based ecological momentary assessment and intervention: bibliometric analysis. Front Psychiatry. (2024) 15:1300739. 10.3389/fpsyt.2024.1300739 38469030 PMC10925651

[59] Fernández-Alvarez J Grassi M Colombo D Botella C Cipresso P Perna G Efficacy of bio- and neurofeedback for depression: a meta-analysis. Psychol Med. (2021) 52(2):201–16. 10.1017/S0033291721004396 34776024 PMC8842225

[60] Weerdmeester J Rooij M Engels RC Granic I. An integrative model for the effectiveness of biofeedback interventions for anxiety regulation: viewpoint. J Med Internet Res. (2020) 22(7):e14958. 10.2196/14958 32706654 PMC7413290

[61] Hickey BA Chalmers T Newton P Lin C Sibbritt D McLachlan CS Smart devices and wearable technologies to detect and monitor mental health conditions and stress: a systematic review. Sensors (Basel). (2021) 21(10):3461. 10.3390/s21103461 34065620 PMC8156923

[62] Gedam S Paul S. A review on mental stress detection using wearable sensors and machine learning techniques. IEEE Access. (2021) 9:84045–66. 10.1109/ACCESS.2021.3085502

[63] Kim H Cheon E Bai D Lee YH Koo B. Stress and heart rate variability: a meta-analysis and review of the literature. Psychiatry Investig. (2018) 15(3):235–45. 10.30773/pi.2017.08.17 29486547 PMC5900369

[64] Weber J Angerer P Apolinário-Hagen J. Physiological reactions to acute stressors and subjective stress during daily life: a systematic review on ecological momentary assessment (EMA) studies. PLoS One. (2022) 17(7):e0271996. 10.1371/journal.pone.0271996 35895674 PMC9328558

[65] Kim H Song J Kim S Lee S Park Y Lee S Recent advances in multiplexed wearable sensor platforms for real-time monitoring lifetime stress: a review. Biosensors. (2023) 13(4):470. 10.3390/bios13040470 37185545 PMC10136450

[66] Moura I Teles A Viana D Marques J Coutinho L Silva F. Digital phenotyping of mental health using multimodal sensing of multiple situations of interest: a systematic literature review. J Biomed Inform. (2023) 138:104278. 10.1016/j.jbi.2022.104278 36586498

[67] Alhejaili R Alomainy A. The use of wearable technology in providing assistive solutions for mental well-being. Sensors. (2023) 23(17):7378. 10.3390/s23177378 37687834 PMC10490605

[68] Mohr DC Zhang M Schueller SM. Personal sensing: understanding mental health using ubiquitous sensors and machine learning. Annu Rev Clin Psychol. (2017) 13(1):23–47. 10.1146/annurev-clinpsy-032816-044949 28375728 PMC6902121

[69] Torous J Kiang MV Lorme J Onnela J. New tools for new research in psychiatry: a scalable and customizable platform to empower data driven smartphone research. JMIR Ment Health. (2016) 3(2):e16. 10.2196/mental.5165 27150677 PMC4873624

[70] Peters MDJ Marnie C Tricco AC Pollock D Munn Z Alexander L Updated methodological guidance for the conduct of scoping reviews. JBI Evidence Synthesis. (2020) 18(10):2119. 10.11124/JBIES-20-00167 33038124

[71] JBI Resources. (Accessed April 9, 2024).

[72] Tricco AC Lillie E Zarin W O'Brien KK Colquhoun H Levac D PRISMA Extension for scoping reviews (PRISMA-ScR): checklist and explanation. Ann Intern Med. (2018) 169(7):467–73. 10.7326/M18-0850 30178033

[73] Jia S Gao H Xue Z Meng X. Recent advances in multifunctional wearable sensors and systems: design, fabrication, and applications. Biosensors (Basel). (2022) 12(11):1057. 10.3390/bios12111057 36421175 PMC9688294

[74] Perez AJ Zeadally S. Recent advances in wearable sensing technologies. Sensors (Basel, Switzerland). (2021) 21(20):6828. 10.3390/s21206828 34696040 PMC8541055

[75] Magal N Rab SL Goldstein P Simon L Jiryis T Admon R. Predicting chronic stress among healthy females using daily-life physiological and lifestyle features from wearable sensors. Chronic Stress (Thousand Oaks). (2022) 6:24705470221100987. 10.1177/24705470221100987 35911618 PMC9329827

[76] Tsujikawa M Kitade T Suzuki K Shibuya K. Accurate chronic stress estimation with personalized models based on correlation maximization. EMBC. (2022) 2022:1761–5. 10.1109/EMBC48229.2022.9871917 36085859

[77] Hirten RP Danieletto M Tomalin L Choi KH Zweig M Golden E Factors associated with longitudinal psychological and physiological stress in health care workers during the COVID-19 pandemic: observational study using apple watch data. J Med Internet Res. (2021) 23(9):e31295. 10.2196/31295 34379602 PMC8439178

[78] Rodrigues S Dias D Aleixo M Retorta A Cunha JPS (eds). Implementing a quantified occupational health sensing platform in the aviation sector: an exploratory study in routine air traffic control work shifts. 2021 43rd Annual International Conference of the IEEE Engineering in Medicine & Biology Society (EMBC) (2021). 10.1109/EMBC46164.2021.9630475 34892752

[79] van Kraaij AWJ Schiavone G Lutin E Claes S Van Hoof C. Relationship between chronic stress and heart rate over time modulated by gender in a cohort of office workers: cross-sectional study using wearable technologies. J Med Internet Res. (2020) 22(9):e18253. 10.2196/18253 32902392 PMC7511872

[80] Schilling R Herrmann C Ludyga S Colledge F Brand S Pühse U Does cardiorespiratory fitness buffer stress reactivity and stress recovery in police officers? A real-life study. Front Psychiatry. (2020) 11:594. 10.3389/fpsyt.2020.00594 32670116 PMC7331850

[81] Timmons AC Han SC Chaspari T Kim Y Pettit C Narayanan S Family-of-origin aggression, dating aggression, and physiological stress reactivity in daily life. Physiol Behav. (2019) 206:85–92. 10.1016/j.physbeh.2019.03.020 30902632 PMC6532643

[82] Nakashima Y Umematsu T Tsujikawa M Onishi Y. An effectiveness comparison between the use of activity state data and that of activity magnitude data in chronic stress recognition. 2019 8th International Conference on Affective Computing and Intelligent Interaction Workshops and Demos (Aciiw) (2019). p. 239–43

[83] Smets E Rios Velazquez E Schiavone G Chakroun I D'Hondt E De Raedt W Large-scale wearable data reveal digital phenotypes for daily-life stress detection. NPJ Digit Med. (2018) 1:67–9; eCollection 2018. 10.1038/s41746-018-0074-9 31304344 PMC6550211

[84] Wilbur RE Griffin JS Sorensen M Furberg RD. Establishing digital biomarkers for occupational health assessment in commercial Salmon fishermen: protocol for a mixed-methods study. JMIR Res Protoc. (2018) 7(12):e10215. 10.2196/10215 30530453 PMC6305878

[85] Berrocal A Katarzyna WAC. Peer-ceived well-being: exploring the value of peers for human stress assessment in-situ. UbiComp/ISWC 2018—adjunct Proceedings of the 2018 ACM International Joint Conference on Pervasive and Ubiquitous Computing and Proceedings of the 2018 ACM International Symposium on Wearable Computers (2018). p. 492–7. 10.1145/3267305.3267319

[86] Kreibig SD Ten Brink M Mehta A Talmon A Zhang J Brown AS The role of emotion regulation, affect, and sleep in individuals with sleep bruxism and those without: protocol for a remote longitudinal observational study. JMIR Res Protoc. (2023) 12:e41719. 10.2196/41719 37616042 PMC10485716

[87] Sharma K Pappas I Papavlasopoulou S Giannakos M (eds). Wearable sensing and quantified-self to explain learning experience. 2022 International Conference on Advanced Learning Technologies (ICALT) (2022).

[88] Schmid RF Thomas J. The interactive effects of heart rate variability and mindfulness on indicators of well-being in healthcare professionals’ daily working life. Int J Psychophysiol. (2021) 164:130–8. 10.1016/j.ijpsycho.2021.01.012 33548348

[89] Juarascio AS Crochiere RJ Tapera TM Palermo M Zhang F. Momentary changes in heart rate variability can detect risk for emotional eating episodes. Appetite. (2020) 152:104698. 10.1016/j.appet.2020.104698 32278643

[90] Williams LM Pines A Goldstein-Piekarski AN Rosas LG Kullar M Sacchet MD The ENGAGE study: integrating neuroimaging, virtual reality and smartphone sensing to understand self-regulation for managing depression and obesity in a precision medicine model. Behav Res Ther. (2018) 101:58–70. 10.1016/j.brat.2017.09.012 29074231 PMC8109191

[91] Vabba A Porciello G Monti A Panasiti MS Aglioti SM. A longitudinal study of interoception changes in the times of COVID-19: effects on psychophysiological health and well-being. Heliyon. (2023) 9(4):e14951. 10.1016/j.heliyon.2023.e14951 37035351 PMC10065811

[92] Plans D Ponzo S Morelli D Cairo M Ring C Keating CT Measuring interoception: the phase adjustment task. Biol Psychol. (2021) 165:108171. 10.1016/j.biopsycho.2021.108171 34411620

[93] Castaldo R Melillo P Bracale U Caserta M Triassi M Pecchia L. Acute mental stress assessment via short term HRV analysis in healthy adults: a systematic review with meta-analysis. Biomed Signal Process Control. (2015) 18:370–7. 10.1016/j.bspc.2015.02.012

[94] Man ISC Shao R Hou WK Xin Li S Liu FY Lee M Multi-systemic evaluation of biological and emotional responses to the trier social stress test: a meta-analysis and systematic review. Front Neuroendocrinol. (2023) 68:101050. 10.1016/j.yfrne.2022.101050 36410619

[95] Giannakakis G Grigoriadis D Giannakaki K Simantiraki O Roniotis A Tsiknakis M. Review on psychological stress detection using biosignals. IEEE Transactions on Affective Computing. (2022) 13(1):440–60. 10.1109/TAFFC.2019.2927337

[96] Can YS Arnrich B Ersoy C. Stress detection in daily life scenarios using smart phones and wearable sensors: a survey. J Biomed Inform. (2019) 92:103139. 10.1016/j.jbi.2019.103139 30825538

[97] Lubitz SA Faranesh AZ Selvaggi C Atlas SJ McManus DD Singer DE Detection of atrial fibrillation in a large population using wearable devices: the fitbit heart study. Circulation. (2022) 146(19):1415–24. 10.1161/CIRCULATIONAHA.122.060291 36148649 PMC9640290

[98] Królak A Pilecka E (eds). Analysis and comparison of heart rate variability signals derived from PPG and ECG sensors. Cham: Springer International Publishing. (2022).

[99] Fernstad J Svennberg E Åberg P Kemp Gudmundsdottir K Jansson A Engdahl J. Validation of a novel smartphone-based photoplethysmographic method for ambulatory heart rhythm diagnostics: the SMARTBEATS study. EP Europace. (2024) 26(4):euae079. 10.1093/europace/euae079 PMC11023506 38533836

[100] Hernando D Roca S Sancho J Alesanco Á Bailón R. Validation of the apple watch for heart rate variability measurements during relax and mental stress in healthy subjects. Sensors (Basel. (2018) 18(8):2619. 10.3390/s18082619 30103376 PMC6111985

[101] Moscato S Palmerini L Palumbo P Chiari L. Quality assessment and morphological analysis of photoplethysmography in daily life. Front Digit Health. (2022) 4:912353. 10.3389/fdgth.2022.912353 35873348 PMC9300860

[102] Quigley KS Gianaros PJ Norman GJ Jennings JR Berntson GG Geus EJC. Publication guidelines for human heart rate and heart rate variability studies in psychophysiology—part 1: physiological underpinnings and foundations of measurement. Psychophysiology. (2024) 61(9):e14604. 10.1111/psyp.14604 38873876 PMC11539922

[103] Pedrelli P Fedor S Ghandeharioun A Howe E Ionescu DF Bhathena D Monitoring changes in depression severity using wearable and Mobile sensors. Front Psychiatry. (2020) 11:584711. 10.3389/fpsyt.2020.584711 33391050 PMC7775362

[104] Fonseka LN Woo BKP. Wearables in schizophrenia: update on current and future clinical applications. JMIR Mhealth Uhealth. (2022) 10(4):e35600. 10.2196/35600 35389361 PMC9030897

[105] Lui GY Loughnane D Polley C Jayarathna T Breen PP. The apple watch for monitoring mental health–related physiological symptoms: literature review. JMIR Ment Health. (2022) 9(9):e37354. 10.2196/37354 36069848 PMC9494213

[106] Ortega MC Bruno E Richardson MP. Electrodermal activity response during seizures: a systematic review and meta-analysis. Epilepsy Behav. (2022) 134:108864. 10.1016/j.yebeh.2022.108864 35952508

[107] Velmovitsky PE Alencar P Leatherdale ST Cowan D Morita PP. Using apple watch ECG data for heart rate variability monitoring and stress prediction: a pilot study. Front Digit Health. (2022) 4:1058826. 10.3389/fdgth.2022.1058826 36569803 PMC9780663

[108] Iqbal SMA Mahgoub I Du E Leavitt MA Asghar W. Advances in healthcare wearable devices. Npj Flex Electron. (2021) 5(1):1–14. 10.1038/s41528-021-00107-x

[109] Sheikh M Qassem M Kyriacou PA. Wearable, environmental, and smartphone-based passive sensing for mental health monitoring. Front Digit Health. (2021) 3:662811. 10.3389/fdgth.2021.662811 34713137 PMC8521964

[110] López-Larraz E Escolano C Robledo-Menéndez A Morlas L Alda A Minguez J. A garment that measures brain activity: proof of concept of an EEG sensor layer fully implemented with smart textiles. Front Hum Neurosci. (2023) 17:1135153. 10.3389/fnhum.2023.1135153 37305362 PMC10250743

[111] Alam NB Surani M Das CK Giacco D Singh SP Jilka S. Challenges and standardisation strategies for sensor-based data collection for digital phenotyping. Commun Med. (2025) 5(1):360. 10.1038/s43856-025-01013-3 40830260 PMC12365157

[112] Henson P D’Mello R Vaidyam A Keshavan M Torous J. Anomaly detection to predict relapse risk in schizophrenia. Transl Psychiatry. (2021) 11(1):1–6. 10.1038/s41398-020-01123-7 33431818 PMC7798381

[113] Onnela Lab. Digital Phenotyping and Beiwe Research Platform (2024). (Accessed June 17, 2024).

[114] Nissen M Slim S Jäger K Flaucher M Huebner H Danzberger N Heart rate measurement accuracy of fitbit charge 4 and samsung galaxy watch Active2: device evaluation study. JMIR Formative Research. (2022) 6(3):e33635. 10.2196/33635 35230250 PMC8924780

[115] Cao R Azimi I Sarhaddi F Niela-Vilen H Axelin A Liljeberg P Accuracy assessment of oura ring nocturnal heart rate and heart rate variability in comparison with electrocardiography in time and frequency domains: comprehensive analysis. J Med Internet Res. (2022) 24(1):e27487. 10.2196/27487 35040799 PMC8808342

[116] King RC Villeneuve E White RJ Sherratt RS Holderbaum W Harwin WS. Application of data fusion techniques and technologies for wearable health monitoring. Med Eng Phys. (2017) 42:1–12. 10.1016/j.medengphy.2016.12.011 28237714

[117] Baltrušaitis T Ahuja C Morency L. Multimodal machine learning: a survey and taxonomy. IEEE Trans Pattern Anal Mach Intell. (2019) 41(2):423–43. 10.1109/TPAMI.2018.2798607 29994351

[118] Beauchaine TP Thayer JF. Heart rate variability as a transdiagnostic biomarker of psychopathology. Int J Psychophysiol. (2015) 98(2, Part 2):338–50. 10.1016/j.ijpsycho.2015.08.004 26272488

[119] Ernst G. Heart-Rate variability—more than heart beats? Front Public Health. (2017) 5 240. 10.3389/fpubh.2017.00240 28955705 PMC5600971

[120] Perna G Riva A Defillo A Sangiorgio E Nobile M Caldirola D. Heart rate variability: can it serve as a marker of mental health resilience? Special section on “translational and neuroscience studies in affective disorders” section editor, maria Nobile MD, PhD. J Affect Disord. (2020) 263:754–61. 10.1016/j.jad.2019.10.017 31630828

[121] Arakaki X Arechavala RJ Choy EH Bautista J Bliss B Molloy C The connection between heart rate variability (HRV), neurological health, and cognition: a literature review. Front Neurosci. (2023) 17:1055445. 10.3389/fnins.2023.1055445 36937689 PMC10014754

[122] Williams DP Cash C Rankin C Bernardi A Koenig J Thayer JF. Resting heart rate variability predicts self-reported difficulties in emotion regulation: a focus on different facets of emotion regulation. Front Psychol. (2015) 6:261. 10.3389/fpsyg.2015.00261 25806017 PMC4354240

[123] Ishaque S Khan N Krishnan S. Trends in heart-rate variability signal analysis. Front Digit Health. (2021) 3:639444. 10.3389/fdgth.2021.639444 34713110 PMC8522021

[124] Lischke A Pahnke R Mau-Moeller A Weippert M. Heart rate variability modulates interoceptive accuracy. Front Neurosci. (2021) 14:612445. 10.3389/fnins.2020.612445 33536870 PMC7849500

[125] Zhang Y Stewart C Ranjan Y Conde P Sankesara H Rashid Z Large-scale digital phenotyping: identifying depression and anxiety indicators in a general UK population with over 10,000 participants. J Affect Disord. (2025) 375:412–22. 10.1016/j.jad.2025.01.124 39892753

[126] Dai R Kannampallil T Kim S Thornton V Bierut L Lu C. Detecting mental disorders with wearables: a large cohort study. | Proceedings of the 8th ACM/IEEE Conference on Internet of Things Design and Implementation. Proceedings of the 8th ACM/IEEE Conference on Internet of Things Design and Implementation (2023). p. 39–51. 10.1145/3576842.3582389

[127] Wyatt B Forstmann N Badier N Hamy A Larochelambert QD Antero J Changes in physical activity, heart rate, and sleep measured by activity trackers during the COVID-19 pandemic across 34 countries: retrospective analysis. J Med Internet Res. (2025) 27(1):e68199. 10.2196/68199 40184182 PMC12008701

[128] Alvarez-Ambrosio M Chausa P Moreno-Blanco D Roca-Ventura A Oropesa I Cattaneo G Digital phenotyping for assessment and prediction of interoception, chronic stress, and self-regulation in adults: a scoping review. JMIR Preprints [Preprint] (2024). Available online at: https://preprints.jmir.org/preprint/70141 (Accessed January 15, 2026). 10.3389/fdgth.2026.1710891 PMC12926408 41737813

