# Supplementary material for: Digital phenotyping for assessment and prediction of interoception, chronic stress, and self-regulation in adults: a scoping review
Source: Front Digit Health. 2026 Feb 9;8:1710891. doi: 10.3389/fdgth.2026.1710891 (PMC12926408; doi:10.3389/fdgth.2026.1710891)
Supplement: Supplementary file 1 [file Datasheet1.docx]

Supplementary Material

# Supplementary Figures and Tables

## Supplementary Tables

Supplementary Table 1. Preferred Reporting Items for Systematic reviews and Meta-Analyses extension for Scoping Reviews (PRISMA-ScR) Checklist

|  | **ITEM** | **PRISMA-ScR CHECKLIST ITEM** | **REPORTED ON PAGE #** |
| --- | --- | --- | --- |
| **TITLE** | | | |
| Title | 1 | Identify the report as a scoping review. |  |
| **ABSTRACT** | | | |
| Structured summary | 2 | Provide a structured summary that includes (as applicable) background, objectives, eligibility criteria, sources of evidence, charting methods, results, and conclusions that relate to the review questions and objectives. | Abstract |
| **INTRODUCTION** | | | |
| Rationale | 3 | Describe the rationale for the review in the context of what is already known. Explain why the review questions/objectives lend themselves to a scoping review approach. | 1.Introduction |
| Objectives | 4 | Provide an explicit statement of the questions and objectives being addressed with reference to their key elements (e.g., population or participants, concepts, and context) or other relevant key elements used to conceptualize the review questions and/or objectives. | 2.1 Review questions |
| **METHODS** | | | |
| Protocol and registration | 5 | Indicate whether a review protocol exists; state if and where it can be accessed (e.g., a Web address); and if available, provide registration information, including the registration number. | 2.1 Review questions |
| Eligibility criteria | 6 | Specify characteristics of the sources of evidence used as eligibility criteria (e.g., years considered, language, and publication status) and provide a rationale. | 2.2 Eligibility criteria |
| Information sources* | 7 | Describe all information sources in the search (e.g., databases with dates of coverage and contact with authors to identify additional sources), as well as the date the most recent search was executed. | 2.2 Eligibility criteria  2.3 Search strategy |
| Search | 8 | Present the full electronic search strategy for at least 1 database, including any limits used, such that it could be repeated. | Supplementary Table 2 |
| Selection of sources of evidence† | 9 | State the process for selecting sources of evidence (i.e., screening and eligibility) included in the scoping review. | 2.2 Eligibility criteria |
| Data charting process‡ | 10 | Describe the methods of charting data from the included sources of evidence (e.g., calibrated forms or forms that have been tested by the team before their use, and whether data charting was done independently or in duplicate) and any processes for obtaining and confirming data from investigators. | 2.5 Data extraction  Supplementary Table 3 |
| Data items | 11 | List and define all variables for which data were sought and any assumptions and simplifications made. | N/A |
| Critical appraisal of individual sources of evidence§ | 12 | If done, provide a rationale for conducting a critical appraisal of included sources of evidence; describe the methods used and how this information was used in any data synthesis (if appropriate). | N/A |
| Synthesis of results | 13 | Describe the methods of handling and summarizing the data that were charted. | 2.5 Data extraction  Supplementary Table 3 |
| **RESULTS** | | | |
| Selection of sources of evidence | 14 | Give numbers of sources of evidence screened, assessed for eligibility, and included in the review, with reasons for exclusions at each stage, ideally using a flow diagram. | 3. Results  Figure 2 |
| Characteristics of sources of evidence | 15 | For each source of evidence, present characteristics for which data were charted and provide the citations. | N/A |
| Critical appraisal within sources of evidence | 16 | If done, present data on critical appraisal of included sources of evidence (see item 12). | N/A |
| Results of individual sources of evidence | 17 | For each included source of evidence, present the relevant data that were charted that relate to the review questions and objectives. | Tables 1, 2, 3  Supplementary Tables 4, 5, 6 |
| Synthesis of results | 18 | Summarize and/or present the charting results as they relate to the review questions and objectives. | 3. Results |
| **DISCUSSION** | | | |
| Summary of evidence | 19 | Summarize the main results (including an overview of concepts, themes, and types of evidence available), link to the review questions and objectives, and consider the relevance to key groups. | 4.1 Principal findings |
| Limitations | 20 | Discuss the limitations of the scoping review process. | 4.2 Current limitations and future directions  4.3 Limitations of the review methodology |
| Conclusions | 21 | Provide a general interpretation of the results with respect to the review questions and objectives, as well as potential implications and/or next steps. | 4.4 Conclusions |
| **FUNDING** | | | |
| Funding | 22 | Describe sources of funding for the included sources of evidence, as well as sources of funding for the scoping review. Describe the role of the funders of the scoping review. | 7. Fundings |

JBI = Joanna Briggs Institute; PRISMA-ScR = Preferred Reporting Items for Systematic reviews and Meta-Analyses extension for Scoping Reviews.

* Where *sources of evidence* (see second footnote) are compiled from, such as bibliographic databases, social media platforms, and Web sites.

† A more inclusive/heterogeneous term used to account for the different types of evidence or data sources (e.g., quantitative and/or qualitative research, expert opinion, and policy documents) that may be eligible in a scoping review as opposed to only studies. This is not to be confused with *information sources* (see first footnote).

‡ The frameworks by Arksey and O’Malley (6) and Levac and colleagues (7) and the JBI guidance (4, 5) refer to the process of data extraction in a scoping review as data charting*.*

§ The process of systematically examining research evidence to assess its validity, results, and relevance before using it to inform a decision. This term is used for items 12 and 19 instead of "risk of bias" (which is more applicable to systematic reviews of interventions) to include and acknowledge the various sources of evidence that may be used in a scoping review (e.g., quantitative and/or qualitative research, expert opinion, and policy document).

N/A: not applicable

*From:* Tricco AC, Lillie E, Zarin W, O'Brien KK, Colquhoun H, Levac D, et al. PRISMA Extension for Scoping Reviews (PRISMAScR): Checklist and Explanation. Ann Intern Med. 2018;169:467–473. [doi: 10.7326/M18-0850](http://annals.org/aim/fullarticle/2700389/prisma-extension-scoping-reviews-prisma-scr-checklist-explanation).

Supplementary Table 2. Search strategy

|  | Keywords |
| --- | --- |
| Digital phenotyping | "digital phenotyp*” OR "digital sensing" OR "passive sensing" OR "passive detection" OR “personal sensing” OR “mobile sensing” OR “passive monitor*” OR "activity recognition" OR "digital footprint" OR "digital trace*" OR "real-world data" OR “ambulatory monitoring” OR “ambulatory assessment” |
| Digital biomarker | “digital biomarker*” OR "digital marker*" OR “behavior* marker*” |
| Devices | smartphone* OR smartwatch* OR wearable* OR wrist-worn OR “fitness tracker*” OR "activity tracker*" |
| Domain | interoception OR interoceptive  “stress reactivity” OR “reactivity to stress” OR “affective reactivity” OR “emotional reactivity” OR “allostatic load” OR “chronic stress”  “daily stressor*” OR “daily life stress” OR “stress in daily life”  (resilience near/4 brain) OR (resilience NEAR/4 stress) OR "psychological resilience"  “self-regulation” OR “emotion regulation” OR “emotional regulation" |
| NOT | adolescent* OR child* OR student*  rat* OR mouse OR mice OR animal* OR monkey* |
| Search strategy  Database |  |
| PubMed | (((("interoception"[Title/Abstract] OR "interoceptive"[Title/Abstract] OR "stress reactivity"[Title/Abstract] OR "reactivity to stress"[Title/Abstract] OR "affective reactivity"[Title/Abstract] OR "emotional reactivity"[Title/Abstract] OR "allostatic load"[Title/Abstract] OR "chronic stress"[Title/Abstract] OR "daily stressor*"[Title/Abstract] OR "daily life stress"[Title/Abstract] OR "self-regulation"[Title/Abstract] OR "emotion regulation"[Title/Abstract] OR "emotional regulation"[Title/Abstract] OR "resilience brain"[Title/Abstract:~4] OR "resilience stress"[Title/Abstract:~4] OR "psychological resilience"[Title/Abstract]) AND ("digital phenotyp*"[Title/Abstract] OR "digital sensing"[Title/Abstract] OR "passive sensing"[Title/Abstract] OR "passive detection"[Title/Abstract] OR "personal sensing"[Title/Abstract] OR "mobile sensing"[Title/Abstract] OR "passive monitor*"[Title/Abstract] OR "activity recognition"[Title/Abstract] OR "digital footprint"[Title/Abstract] OR "digital trace*"[Title/Abstract] OR "real-world data"[Title/Abstract] OR "ambulatory monitoring"[Title/Abstract] OR "ambulatory assessment"[Title/Abstract] OR "digital biomarker*"[Title/Abstract] OR "digital marker*"[Title/Abstract] OR "behavior marker"[Title/Abstract] OR "smartphone*"[Title/Abstract] OR "smartwatch*"[Title/Abstract] OR "wearable*"[Title/Abstract] OR "wrist-worn"[Title/Abstract] OR "fitness tracker*"[Title/Abstract] OR "activity tracker*"[Title/Abstract])) NOT ("adolescent*"[Title/Abstract] OR "child*"[Title/Abstract] OR "student*"[Title/Abstract] OR "rat"[Title/Abstract] OR "mouse"[Title/Abstract] OR "mice"[Title/Abstract] OR "animal*"[Title/Abstract] OR "monkey*"[Title/Abstract])) NOT (("meta analysis"[Publication Type] OR "review"[Publication Type] OR "systematic review"[Filter]) AND 2018/01/01:3000/12/12[Date - Publication])) AND (2018/1/1:3000/12/12[pdat]) |

Supplementary Table 3. Data extraction form

| Study citation |  |
| --- | --- |
| **Study details** |  |
| Title |  |
| Authors |  |
| Year of publication |  |
| Country |  |
| Aims/purpose |  |
| Method |  |
| Sample size |  |
| Study population (age, sex …) |  |
| Study design |  |
|  |  |
| Domain |  |
| Proxies, targets |  |
| Study duration |  |
| Devices |  |
| Sensors |  |
| Smartphone |  |
| Measures \|data acquisition |  |
| Technological platform/app |  |
| Procedure |  |
| EMA |  |
| Sensor data pre-Processing |  |
| Data preprocessing and Feature extraction |  |
| Data analyst\| Analytical method |  |
| Self-report questionnaires |  |
| Source of validation |  |
| Key finding |  |

**Supplementary Table 4**. Summary of the information collected from the studies involving measures of interoception, including the interoceptive dimension explored, the tasks or passive data utilized to assess interoception, the measurement conditions, as well as the low-level features and core analytical approach used for data processing (Statistics, Machine Learning, Hybrid).

| **Study reference** | **Interoceptive dimension**  **Variables** | **Task /Passive data - measurement condition** | **Low-level features** | **Active data** | **Core analytical approach / Model** |
| --- | --- | --- | --- | --- | --- |
| Plans et al^a^ [92], 2023 | Interoceptive accuracy | Phase Adjustment Task / heartbeats-  1 session | Phase Adjustment Task results |  | Hybrid (ML + Stats)  Gaussian Mixture Models (GMM) + Bayes Factor |
|  | Interoceptive awareness |  |  | 1 item questionnaire |  |
|  | Interoceptive sensibility |  |  | Online platform: Body Perception Questionnaire (BPQ) - Awareness subscale of the Interoceptive Accuracy Scale |  |
|  | Physiological parameters | heartbeats -  2-minute session | HR, HRV (SDNN RMSSD, pNN50) |  |  |
| Vabba et al [91], 2021 | Interoceptive accuracy | Heartbeat Counting Task /heartbeats  3 sessions^b^ | Heartbeat Counting Task results | 1 item questionnaire | Statistics  Linear Mixed-Effects Models (LMM) + Hierarchical Regression |
|  | Interoceptive awareness |  |  |  |  |
|  | Interoceptive sensibility |  |  | Online platform: Multidimensional Assessment of Interoceptive Awareness (MAIA), Body Perception Questionnaire (BPQ) |  |
|  | Physiological wellbeing^c^ | Heartbeats -  5- minute session | HRV (RMSSD) |  |  |
| ^NOTES:^  ^a^ The paper by Plans et al comprises two studies but only the first study is included in this review.  ^b^ The study lasted one year, with interoception-related measures collected at three time points: one laboratory session and two ambulatory sessions.  ^c^ Indexed by HRV. | | | | | |

**Supplementary Table 5.** Summary of the information collected from the studies involving measures of stress (chronic stress and stress reactivity), including the active and passive data used, the measurement condition (how the wearable device is used), low-level features, and core analytical approach used for data processing (Statistics, Machine Learning, Hybrid) (if applicable).

| **Study reference** | **Domain** | **Passive data** | **Measurement conditions** | **Active data** | **Low level features** | **Core analytical approach / Model** |
| --- | --- | --- | --- | --- | --- | --- |
| Magal et al [75], 2022 | Chronic stress | HR, sleep measures, steps, altitude and minutes spent in different activity levels^a^ | Continuous wear in daily life | Baseline: Trier Inventory for Chronic Stress  No EMA | HR statistical measures, HR cosinor metrics, sleep and activity features  BMI, age, smoking status and Normalized Difference Vegetation Index (NDVI) | Machine Learning (Classification)  Support Vector Machine (SVM) with Polynomial Kernel |
| Tsujikawa et al [76], 2022 | Chronic stress | EDA, ACC^b^ | Worn during work hours | Baseline and once a month: PSS  No EMA | EDA features | Hybrid (ML + Optimization)  k-means Clustering + Correlation Maximization-based Classification |
| Rodrigues et al [78], 2021 | Chronic stress | ECG | Discontinuous, during shifts and days off | Baseline: PSS  EMA: daily stress symptoms questionnaire | HRV time domain metrics (AVNN, RMSSD pNN50, LF/HF) | Statistics  Wilcoxon Signed-Rank Test + Kruskal-Wallis Test |
| Hirten et al [77], 2021 | Chronic stress | HRV^c^ | Continuous wear in daily life^d^ | Baseline: PSS-4  EMA: weekly PSS-4 | HRV circadian metrix (cosinor model)  gender, age, occupation, baseline resilience, optimism, and quality of life | Statistics  Linear Mixed-Effects Models (LMEM) with First-Order Autoregressive (AR) Correlation |
| van Kraaij et al [79], 2020 | Chronic stress | ECG | Continuous wear in daily life^e^ | PSS-10 (via web)  No EMA | HR, HR circadian harmonics  weekend (yes/no), gender, age | Statistics  Linear Mixed-Effects Model (LMM) |
| Schilling et al [80], 2020 | Stress reactivity | ECG | Continuous wear in daily life | Baseline: Effort-Reward Imbalance scale, Job Demand and Control scaleEMA: (feelings of stress 1 item) | HRV (RMSSD) | Statistics  Multilevel Modeling (MLM) + Hierarchical Regression |
| Timmons et al [81], 2019 | Stress reactivity | EDA | Continuous wear in daily life | EMA | SC level | Statistics  Multilevel Structural Equation Modeling (MLM-SEM) |
| Nakashima et al [82], 2019 | Chronic stress | EDA, ACC ^f^ | Worn during work hours | PSS-10  EMA: no | EDA features | Machine Learning (Regression)  Linear Support Vector Regression (SVR) |
| Smets et al [83], 2018 | Stress reactivity | ECG , ACC ^f^, SC, ST  smartphone's virtual and physical sensor data^g^ | Continuous wear in daily life | Baseline: Depression, Anxiety Stress Scale -, PSS, Montreal Imaging Stress Task  EMA (12 times per day 4 items related with levels of stress) | mean HR, HRV time and frequency indices (SDNN, RMSSD, LF, HF, LF/HF) , SC features, ST features, ACC std  Smartphone features: location, usage,  audio, movement, SMS/call/mail logs, and features from environmental sensors (ambient light, air pressure, temperature), screen mode (on/off)) | Hybrid (ML + Stats)  Random Forest Classifier + Linear Mixed-Effects Models (LMEM) |
| Wilbur et al [84], 2018 | Chronic stress  (work-related) | ECG, respirations, ACC | Continuous wear in daily life | No | HRV, respiratory rate, physical activity index  salivary cortisol  Observational data collected by the primary investigator | Statistics  Mixed-Model Trajectory Analysis (LMM) |
| (Berrocal & Katarzyna [85], 2018 | Chronic stress | HRV | Twice a day wake-up and bedtime | Baseline: PSS, custom survey about stress awareness  EMA: perceived stress | HRV  Smartphone usage statistics | Machine Learning (Classification)  ML Classification Model |
| NOTES  a The study uses the data preprocesses by Fitbit.  b The ACC data is used to estimate subject activity states, but are noy utilized in the models.  ^c^ It is provided directly by the device's app (Apple Watch).  d Participants were instructed to wear the Apple Watch for at least 8 hours per day throughout the study period.  e Participants were asked to remove the chest patch during vigorous physical activities and to shower with a protective cover.  ^f^ The ACC data is used to consider the influences of physical activity on the physiological signals.  g Smartphone contextual data, usage, movement, environmental sensors. | | | | | | |

Supplementary Table 6. Summary of the information collected from studies involving measures of self-regulation, including the active and passive data used, the measurement condition (how the wearable device was used), low-level features and the core analytical approach used for data processing (Statistics, Machine Learning, Hybrid). (if applicable).

| **Study reference** | **Domain** | **Passive data** | **Measurement condition** | **Active data** | **Low level features** | **Core analytical approach / Model** |
| --- | --- | --- | --- | --- | --- | --- |
| Kreibig et al [86], 2023 | Emotion regulation | ECG | Continuous wear in daily life | Baseline: Emotion Regulation Questionnaire EMA | HRV | N/A^a^ |
| K. Sharma et al [87], 2022 | Emotion regulation | IBI^f^ | During class sessions | No | HRV | N/A^a^ |
| Schmid & Thomas [88], 2021 | Emotion regulation | ECG | During shift work | No | HRV (RMSSD) | N/A^a^ |
| Juarascio et al [89], 2020 | Emotion regulation | IBI | Waking hours | No | HRV metrics at time and frequency domain (SDNN, SDANN, SDNNIX pNN50, SDSD, RMSSD, IRRR, MADRR, TINN; the mean Low Frequency signal, the mean High Frequency signal);  HR ( mean night HR, mean HR) | N/A^a^ |
| Williams, L. M. et al [90], 2018 | Self-regulation | Smartphone usage data, GPS | Continuous wear in daily life | In lab: Emotion Regulation Questionnaire; the brief COPE; The Brief Risk-resilience Index for Screening | Smartphone use features (word frequencies collected from emails, text messages or search terms; punctuation usage; incoming and outgoing calls and messages; typing latencies, reaction times, phone stimuli detection), activity | Hybrid (ML + Stats)  Sparse Regression  Hierarchical Clustering  Classification +  Linear Regression, Correlation |
| ^a^ N/A: not applicable. The study did not involve predicting self-regulation nor search for new self-regulation biomarkers. | | | | | | |

Supplementary Table 7. Abbreviations

| **Abbreviation** | **Definition** |
| --- | --- |
| ACC | Accelerometer data |
| AVNN | Average of NN intervals |
| BMI | Body mass index |
| BRISC | The Brief Risk-resilience Index for Screening |
| ECG | Electrocardiogram data |
| EDA | Electrodermal activity |
| EMA | Ecological momentary assessment |
| ERQ | Emotion Regulation Questionnaire |
| HF (HF1, HF2, … HF6) | The mean High Frequency signal |
| HR | Heart rate |
| HRV | Heart rate variability |
| IBI | Inter beat interval |
| IRRR | Length of the interval determined by the first and the third quantile of the inter-beat interval |
| LF/HF | Ratio of LF (low frequency) and HF (high frequency) power bands |
| MADRR | Median of the absolute values of the successive differences between the inter-beat intervals |
| Mean HR | Mean interpolated Heart Rate |
| Mean niHR | Mean non-interpolated heart rate |
| ML | Machine learning |
| pNN50 | Percentage of successive NN intervals that differ by more than 50 ms |
| PSS | Perceived stress scale |
| RMSSD | Root mean square of the successive differences |
| SC | Skin conductance |
| ST | Skin temperature |
| SDANN | Standard deviation of NN intervals between all successive heartbeats |
| SDNN | Standard deviation of NN intervals |
| SDNNIX | Mean of the standard deviations of all the filtered inter-beat intervals for each 5 min segment of the IBI stream |
| SDSD | Standard deviation of successive differences of inter-beat intervals |
| SVR | Support vector regression. |
| TINN | Triangular interpolation of NN interval histogram |
